# A coupled forage-grazer model predicts viability of livestock production and wildlife habitat at the regional scale

**DOI:** 10.1038/s41598-019-56470-3

**Published:** 2019-12-30

**Authors:** Virginia A. Kowal, Sharon M. Jones, Felicia Keesing, Brian F. Allan, Jennifer M. Schieltz, Rebecca Chaplin-Kramer

**Affiliations:** 1 Natural Capital Project, Stanford University, Stanford, CA USA; 2 Dept. of Entomology, University of Illinois Urbana-Champaign, Urbana, IL USA; 3 Program in Biology, Bard College, Annandale-on-Hudson, NY USA; 4 Dept. of Ecology & Evolutionary Biology, Princeton University, Princeton, NJ USA

**Keywords:** Conservation biology, Ecological modelling, Grassland ecology, Ecosystem services

## Abstract

Informed management of livestock on rangelands underpins both the livelihoods of communities that depend on livestock for sustenance, and the conservation of wildlife that often depend on livestock-dominated landscapes for habitat. Understanding spatial patterns of rangeland productivity is therefore crucial to designing global development strategies that balance social and environmental benefits. Here we introduce a new rangeland production model that dynamically links the Century ecosystem model with a basic ruminant diet selection and physiology model. With lightweight input data requirements that can be met with global sources, the model estimates the viability of broad livestock management decisions, and suggests possible implications of these management decisions for grazing wildlife. Using minimal field data, the new rangeland production model enables the reliable estimation of cattle stocking density; this is an important predictor of the viability of livestock production and forage available for grazing wildlife.

## Introduction

Rangelands cover 30% of the earth’s ice-free surface and provide nourishment for 800 million people suffering from food insecurity^1^. Rangelands also provide critical habitat for wildlife, especially in countries like Kenya where protected areas contain a minority of the country’s wildlife^2^. Overgrazing of rangelands worldwide by domestic livestock is linked to erosion, desertification, and habitat degradation for the many other species that thrive on healthy grasslands^3^. Sustainable management of the world’s rangelands is therefore crucial to balancing human well-being with ecosystem health and wildlife conservation, but we lack accessible tools for exploring how changes in management could affect social and environmental benefits.

Critical uncertainties also exist about productivity on the world’s rangelands under climate change. As the global climate shifts, rainfall patterns are expected to change both in total amount and in variability^4^. Non-equilibrium theory predicts that variability in precipitation, in addition to total rainfall, strongly limits rangeland productivity^5^, and retrospective analyses support this prediction^6^. Understanding how changing rainfall amount and distribution will impact livestock productivity in the future is crucial to climate change adaptation.

Rangeland ecosystem models are a vital tool for forecasting and understanding productivity changes under climate change^7^, and are a well-established means of assessing management strategies that balance consumption of forage resources with efficient production of livestock^8,9^. Most currently available livestock production models, however, fall on one of two ends of a spectrum of system scale and data requirements^10^. For example, detailed, management-oriented models such as SPUR^11^ and IFSM^12^, while very powerful tools in contrasting ranch-scale management scenarios, require intensive parameterization and are often designed for use in a restricted geography (e.g., the western United States). At the opposite extreme are continental (e.g.^13^) or global (e.g.^1,14,15^) rangeland models. These tools, while useful for monitoring and large-scale forecasting, are limited in the level of detail with which management scenarios can be compared.

Here we introduce a new approach that bridges these two extremes: a dynamic model that preserves much of the complexity of management-oriented models while addressing grazer-forage interactions at a landscape scale. We have designed the model to rely on global data sources and to be readily applicable to decision contexts around the world, similar to the ecosystem services models in the InVEST model suite^16^. Here we use an array of properties managed for cattle production and wildlife conservation in the semi-arid savanna of Laikipia, Kenya as a case study to examine the viability of livestock production and forage for wildlife under variable precipitation scenarios and at different livestock densities. Laikipia is a large (9666 km^2^) region that spans a wide range of rainfall and productivity; there, livestock ranching and wildlife-related tourism are of primary economic importance^17^.

We used field data describing biomass and animal densities in Laikipia (data available from Mendeley Data: 10.17632/4m3xybvdb6.2) to test the new model’s ability to address three key questions: first, can livestock management patterns, which are often unknown at landscape scales, be estimated from minimal field measurements? Second, what are the impacts of magnitude and variability of precipitation on the viability of livestock production? And finally, after accounting for forage offtake by livestock, what is the likely range of grazing wildlife densities that may also be supported under extreme climate and management scenarios?

## Materials and Methods

### Model description

A brief model description is included here; see Supplementary Appendix S2 for full model description and equations.

**Figure 1:**
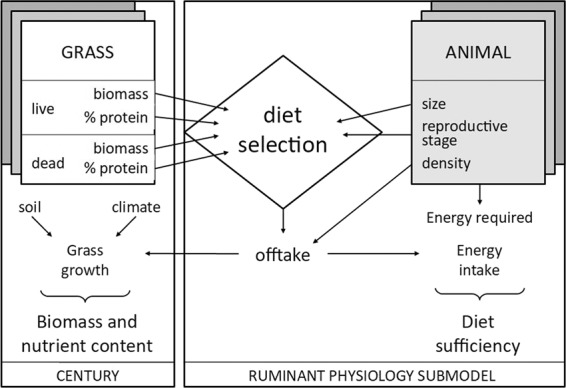
Beta Rangeland Production model overview, showing coupled integration of the Century model (left), which predicts forage growth and nutrient cycling, with the ruminant physiology model adapted from GRAZPLAN (right), which predicts diet selection and diet sufficiency.

The purpose of the beta Rangeland Production model (hereafter, “Rangeland model”) is to project forage production and diet sufficiency for grazing animals under different conditions of climate and management. The structure of this model couples the Century ecosystem model (version 4.6^18^) with a basic ruminant physiology model adapted from GRAZPLAN^19^ to estimate the potential of a given site to meet the forage requirements of ruminant livestock and to support populations of grazing wildlife (Fig. 1). The Century submodel simulates the growth of herbaceous forage according to climate and soil conditions at a monthly timestep. The ruminant physiology submodel adapted from GRAZPLAN calculates offtake of forage by grazing animals according to the biomass and protein content of the simulated forage, and estimates the adequacy of the diet to meet the animals’ energy requirements. Then, the estimated offtake by animals is integrated into the regrowth of forage in the following timestep through impacts on simulated potential production, root:shoot ratio, and plant nitrogen content according to Century’s existing grazing routine^20^.

Inputs to the Rangeland model therefore include all required inputs to Century and descriptive parameters of the modeled livestock herd (Table 1; see Supplementary Appendix S2 for full inputs required). Outputs of the model consist of monthly time series of forage biomass and protein content, forage intake by grazing animals, and an estimate of the adequacy of the forage consumed by animals to meet their maintenance energy requirements, over the same time period as the inputs. The core equations of the animal submodel, which describe forage intake, energy and protein supplied by the diet, and energy and protein requirements for maintenance, were adapted from GRAZPLAN to relate to forage biomass and quality predicted by Century (see Supplementary Appendix S2 for full animal submodel equations). The livestock herd is simulated as a static collection of age/sex classes, since uncertainty about the exact herd demographics over a landscape makes tracking the growth and consequent change in feeding requirements of an individual unrealistic (Supplementary Appendix S1).

**Table 1:** Abbreviated inputs to the beta Rangeland production model.

Input category	Inputs
Soil	Percent sand, silt, clay
	Bulk density
	pH
Climate	Monthly temperature and precipitation
Grass	Century parameter set
	Digestibility or crude protein content (optional)
Livestock (each age/sex class)	Breed, age, weight
	For breeding females: average conception month, average calving interval, average lactation duration
	Stocking density
Site	e.g., latitude, area

For full model description and input list, see Supplementary Appendix S2.

While many existing ranch-level management models track livestock weight, meat, and milk production explicitly, the data required to calibrate these quantities constitute a heavy burden for users, especially at landscape scales, and are beyond the scope of a model that is intended to be broadly applicable. Instead, the Rangeland model focuses on the impact of broad management decisions, such as stocking density and duration, on the viability of livestock production, using the metric of diet sufficiency. This metric describes, for each modeled timestep, the extent to which energy intake of the animal diet meets or exceeds maintenance energy needs. It is calculated as in Eq. (1).

1 $$dietsufficiency=\frac{M{E}_{intake}-M{E}_{req}}{M{E}_{req}}$$

where *ME*_*intake*_ is the total intake of metabolizable energy from the diet and *ME*_*req*_ is energy requirements of maintenance.

A diet sufficiency value greater than zero indicates that the herd is expected to gain weight; a negative value indicates that the diet is insufficient to maintain current weight or condition and the herd is expected to be losing weight.

The model relies on a “management threshold” that must be defined by the user as the minimum residual biomass required to be left standing after forage offtake by livestock. In the case where demand for forage by the herd is predicted to violate the management threshold (i.e., predicted forage offtake by animals would leave very little residual biomass), the model simulates a management decision to halt grazing at the site when the threshold is reached. In reality, the management choices available in cases of very low forage availability are highly manager- and context-specific: some managers would address such a shortfall in available forage by relocating their animals to a site with greater forage availability, while others may choose to import supplemental feed. In the Rangeland model, a shortfall in forage intake restricted by the management threshold is simply reflected by a low diet sufficiency value, indicating that available forage at the simulated site did not meet the animals’ energetic needs.

Note that restriction of forage intake by the management threshold is distinct from, and operates in addition to, the predicted behavioral and physiological response of livestock to forage quantity and quality (see “Model integration”, below, and Supplemental Appendix S2). The management threshold is required because the behavior of the Century model with complete removal of aboveground biomass is undefined. For the demonstrations described here, we estimated the management threshold from the minimum total biomass standing recorded in field surveys.

### Model integration

The two submodels are linked dynamically through herbivore diet selection, a submodel that was adapted from GRAZPLAN but draws its inputs from forage availability and protein content as predicted by Century (Fig. 1, Table 1). The forage resource is modeled as one or several plant species or functional types, which we refer to in the following description as forage types. Each forage type is modeled separately by Century and is characterized by the biomass of its live and standing dead fractions, which fluctuate at each time step according to growth and consumption by herbivores. For the model applications described in the current study, we modeled one forage type only (tropical grass).

At each time step, the forage resource is summarized from Century outputs and supplied as input to the livestock diet selection routine. The relevant descriptors of available forage for the diet selection submodel are biomass and digestibility of live and standing dead fractions of each forage type; this information is collected directly from Century outputs. Diet selection, whereby herbivores select how much of live and dead biomass of each forage type to eat, then proceeds on the available forage. Diet selection from available forage is influenced both by biomass and digestibility of each forage type and by size and condition of the animal (i.e., body mass relative to expected mass for the animal’s breed, sex, age, and reproductive status; see detailed model description in Supplementary Appendix S2).

### Back-calculate management routine

One of the main requirements for tracking rangeland production over time is the grazing management or density of grazers on the land. Like many models that include grazing animals, Century requires as input a management file consisting of scheduled grazing events (months when there were herbivores present) where each grazing event is described by its intensity level (percent biomass removed by herbivores). However, this information is seldom known across landscapes. For this reason, we use a new method to “back-calculate” the management, developing a routine that calibrates the management schedule based on empirical measurement of biomass.

In particular, Century requires a “spin-up” period to establish initial soil nutrient pools, and a management schedule must be supplied for this period. If it is difficult to derive a current or recent management schedule in many natural grasslands, reconstructing a long-term management history of such areas in the detail that the model requires is even less plausible. Therefore, we use the *back-calculate management* routine to set this historical management schedule, based on current observed conditions. While this method assumes that current management is reflective of historic management, in absence of other information to the contrary, it is the simplest way to initialize the Century model.

The *back-calculate management* routine launches the Rangeland model with a starting simulated grazing schedule, compares simulated biomass to an empirical measurement, and iteratively modifies the simulated grazing schedule until empirical biomass is matched. The routine modifies the grazing schedule either by adding or removing grazing events, modifying the intensity of grazing events (i.e., the percent of standing biomass removed by herbivores), or both. If the user specifies that both the schedule and intensity should be modified, the routine first modifies the schedule until no more opportunities exist to do so, and then modifies intensity. For example, if simulated biomass is higher than empirical biomass, the routine adds grazing events prior to the empirical measurement date. If grazing events are added to every month within the maximum amount of time allowable but simulated biomass is still too high, the routine then increases grazing intensity. Intensity is modified at each iterative step of the routine until empirical biomass is matched or the maximum allowable iterations are completed.

### Methods for model testing and application

By coupling two existing models (Century to estimate forage production and an adaptation of GRAZPLAN to predict animal forage offtake and energy requirements), the Rangeland model is intended to be readily applied to new sites that span the wide range of environments where Century and GRAZPLAN have been used and validated (e.g.^18,19,21,22^ for Century^23^; and references therein for GRAZPLAN). In the following sections, we describe testing and application of the Rangeland model to a grassland case study in Kenya.

We conducted the case study simulations to demonstrate the model’s usefulness and test its accuracy in Laikipia County, Kenya, on 25 individually-managed properties which vary in the abundance and co-occurrence of both domestic and wild grazers (Supplementary Fig. S1). Livestock on the majority of the properties are grazed in small herds, continually accompanied by herders, and are sheltered in portable bomas or paddocks at night. On two of the properties, livestock roam freely in large paddocks. Thus, the properties in the study represent a range of livestock management approaches spanning the continuum of common management practices in this semi-arid ecosystem. For a detailed description of ownership and management practices on the 25 properties, see Keesing *et al*.^17^.

First, we tested the model’s ability to infer management by comparing grazing intensity, as calculated by the *back-calculate management* routine, to grazer densities empirically estimated via dung transects. Second, we explored the relative impacts of total precipitation and rainfall variability on livestock production, using the Rangeland model to calculate the maximum viable livestock density at each property in a given year with systematically manipulated rainfall inputs. Finally, we demonstrated the use of the model to predict spatial variability in potential livestock densities and remaining forage available for wildlife across the 25 properties under climate and management change scenarios. For additional detail on model inputs, field data collection, validation of sub-models, and validation of the *back-calculate management* routine, see Supplementary Appendix S1.

#### Using the *back-calculate management* routine to infer grazing pressure

We tested the ability of the *back-calculate management* routine to infer grazing pressure by comparing modeled grazing intensity, as calculated by the routine, to empirical livestock and wildlife densities at eight points across one property, Ol Pejeta Conservancy (OPC; Supplementary Fig. S1). OPC is an integrated property, managed for both livestock and wildlife (*cf  Keesing et al.*^17^), where livestock are grazed in small herds by herders and kept in moveable bomas or paddocks at night. We ran a separate simulation at each of eight weather stations (hereafter, “site”), using a single empirical biomass measurement taken near that site as the biomass target that the routine should match (Supplementary Appendix S1). We quantified the improvement in model fit after estimation of management history by the *back-calculate management* routine with the change in the mean squared error of prediction (ΔMSEP, Eq. (2)^24^). We evaluated any systematic bias of the model in estimating biomass from recent grazing management with the mean bias (Eq. (3)^24^).

2 $$\Delta MSEP=\frac{{\sum }_{i=1}^{n}[{({O}_{i}-{P}_{i,default})}^{2}-{({O}_{i}-{P}_{i,calc})}^{2}]}{n}$$

3 $$MB=\frac{{\sum }_{i=1}^{n}({O}_{i}-{P}_{i,calc})}{n}$$

where *O*_*i*_ is the *i*th observed biomass value; *P*_*i*,*default*_ and *P*_*i*,*calc*_ are the *i*th simulated biomass values using the default and the back-calculated management schedule, respectively; and *n* is the total number of biomass measurements.

We calculated modeled grazing intensity for each site as the average monthly biomass removed by herbivores over the 12 months preceding the final empirical biomass measurement. Empirical animal density surrounding each site was calculated as the average density of dung per transect across transects within the 1600 ha surrounding the site, spanning up to 13 months of field data collection. We classified animals into broad functional groups according to their feeding strategy, and compared the density of each functional group to modeled grazing intensity with simple linear correlation.

#### Regional patterns of livestock production viability and precipitation impacts

Following our test of the Rangeland model at the local scale on OPC, we extended our application of the model to the regional scale to contrast viable levels of livestock production across 25 properties in Laikipia (Supplementary Fig. S1). To describe the relationship between site productivity and livestock production viability, we tracked the number of months in which the livestock herd’s forage intake was insufficient to meet maintenance energy requirements (i.e., number of months with diet sufficiency < 0). We considered this metric an indicator of the viability of livestock production for the given livestock density, for the given site and precipitation inputs.

We ran the model for 24 months following the spin-up period, at a series of stocking density levels spanning the range of reported densities in the region (0.07–1.33 cattle/ha^17^). See Supplementary Appendix S1 for full model inputs. We estimated the management threshold (the residual biomass required to be left standing after diet selection by the herd) as the lowest total standing biomass recorded in field surveys across the region, 300 kg/ha.

We explored the consequences of precipitation change for viable livestock densities in the region, using the Rangeland model to calculate the maximum viable density for each property under manipulated precipitation regimes. We considered the maximum viable density for a given precipitation regime and property to be the density at which the livestock herd experienced one month of diet insufficiency out of the 24-month simulation. We calculated this density by running the model with a first-estimate density and iteratively adjusting the density up and down according to the number of months of diet sufficiency experienced in the previous run. Note that this definition of maximum viable density is subjective, as some livestock managers may tolerate several months of diet insufficiency as “viable”.

For a subset of ten properties, selected randomly to span the range of livestock densities observed in the region, we manipulated precipitation inputs to the model in two ways. First, we changed total annual precipitation by increasing or decreasing precipitation in each month from 20% of its original value up to 120% of the monthly value. Second, we modified the intra-annual distribution of precipitation, independent of total precipitation, by shifting the distribution of precipitation between months. We used the precipitation concentration index (PCI^25^) to compare intra-annual precipitation variability between precipitation scenarios. The PCI is calculated as in Eq. (4).

4 $$PC{I}_{annual}=\frac{{\sum }_{i=1}^{12}{p}_{i}^{2}}{{({\sum }_{i=1}^{12}{p}_{i})}^{2}}\times 100$$

where *p*_*i*_ is monthly precipitation.

PCI ranges from 0 to 100; PCI values < 11 generally are described as having uniform, 11–16 moderate, 16–20 irregular, and >20 strongly irregular precipitation distribution^25^. Simultaneous with perturbations in mean annual precipitation, we shifted the distribution of precipitation within the year to obtain PCI values from 20% to 120% of the empirical value for the site.

In total, we generated 36 precipitation scenarios for each property (i.e., 6 levels of total precipitation in combination with 6 levels of PCI).

Manipulated precipitation inputs were used to drive the “spin-up” simulations in addition to the experimental simulations that lasted for 24 months. In all cases, precipitation did not vary between years of the spin-up simulation or the experimental period - only intra-annual precipitation was varied. We analyzed the relationship of maximum viable density to site and precipitation factors with multiple linear regression, where the response variable was maximum viable density for a given property and precipitation scenario. Predictor variables were the property, total precipitation, PCI, and a two-way interaction including total precipitation and PCI.

#### Estimated viable density of grazing wildlife

We estimated the potential for these properties across Laikipia to support wild grazers after offtake of forage by cattle, given a certain precipitation regime and cattle stocking density, with a simplified index: viable wild grazer density. We calculated viable wild grazer density as the difference between maximum viable cattle density, as estimated by the Rangeland model, and reported cattle density. See Supplementary Table S2 for a table of values that illustrate the metric. Because the Rangeland model integrates the impacts of grazing on forage regrowth, this metric is a more nuanced estimate of the density of wild grazers that could be supported after cattle offtake than strict forage availability *per se*. The index is a meaningful relative metric of additional grazing animals that could be supported by available forage after cattle offtake; the index is expressed as cattle density and is not directly interpretable as number of any specific wildlife species.

We first calculated viable wild grazer density on each property under current livestock stocking density by comparing the maximum viable livestock density to the reported livestock density on that property. We characterized the apparent effect of property management strategy on viable wild grazer density by contrasting the proportion of viable density available for wildlife (i.e., 1 - reported density/maximum viable density) among properties classified by Keesing *et al*.^17^ as primarily dominated by livestock, by wildlife, or by integration of livestock with wildlife.

We then contrasted impacts of extreme regional changes in climate and livestock management on projected viable wild grazer density by repeating the analysis under two climate scenarios and two management scenarios. We calculated the change in viable wild grazer density on each property that would be expected under extreme drought conditions (total annual rainfall decreased to 40% of its current average value), and in conditions of heavily increased rainfall (annual rainfall increased to 160% of its current average value), assuming that the livestock density at each property remained constant. These extremes define the range of total rainfall observed in Laikipia between 1984 and 2014, and are reasonable bounds for climate extremes in the future^26^.

To calculate the potential change in wildlife habitat under region-wide management changes, and to reveal how management extremes compare to climate extremes in terms of their potential to impact wildlife, we estimated viable wild grazer densities under a scenario where each property in the region switched to the lowest regional reported livestock density (0.07 cattle/ha), and to the highest regional reported livestock density (0.43 cattle/ha, after one extreme outlier was removed). These two scenarios therefore describe the range of consequences for regional wildlife habitat if all managers were to switch to a “wildlife-dominated” management strategy, or to a “livestock-dominated” strategy^17^.

## Results

### Modeled *vs*. empirical grazing intensity: how well can we infer grazing management patterns

The *back-calculate management* routine successfully matched target biomass for all eight sites corresponding to weather stations on OPC, and greatly improved the goodness of fit to empirically measured biomass relative to the default management history for northern Kenya (ΔMSEP = 1,188,537 kg/ha; Supplementary Fig. S2). The mean bias of simulated biomass estimates after calibrating grazing intensity was −5.84 kg/ha, indicating that the calibrated model tended to slightly overestimate biomass at the empirical sampling date.

**Figure 2:**
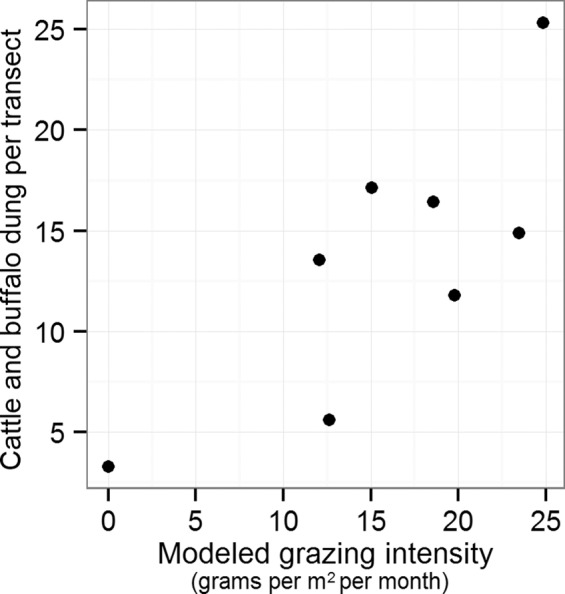
Modeled grazing intensity at eight weather stations on OPC calculated via the *back-calculate management* routine, compared to empirical density of cattle and African buffalo calculated from dung transects. Grazing intensity was calculated from back-calculated schedules as the average monthly biomass removed by herbivores over 12 months preceding the biomass target match date, while dung was averaged across transects near the weather station collected in up to 13 months of sampling.

Average monthly offtake over the 12 months preceding the empirical measuring date ranged from 0 to 248 kg/ha across sites; average percent of total vegetation removed per month varied from 0 to 24.9%. This back-calculated monthly offtake in back-calculated schedules was significantly correlated with the density of dung from cattle and African buffalo (Fig. 2; ρ = 0.79; p = 0.02). Dung density for other functional groups was not correlated with modeled grazing intensity (Supplementary Table S1).

### Regional and temporal variability in precipitation: what are the impacts on livestock production viability

The number of months in which the herd’s forage intake was insufficient to meet maintenance needs, and hence for livestock production to operate viably at given densities, was variable among sites and was strongly related to annual rainfall (Supplementary Fig. S3). As expected, the herd was more vulnerable at higher densities, meaning there were more months at the high densities where the herd was unable to meet its energy needs.

**Figure 3:**
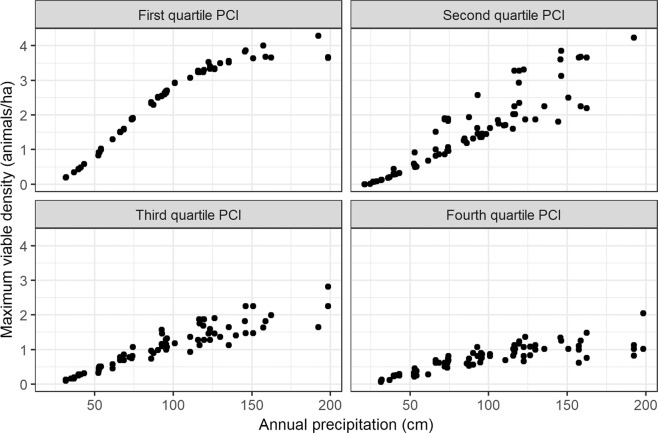
Relationship between annual precipitation and maximum viable livestock density, within quartile ranges of intra-annual precipitation as described by the precipitation concentration index (PCI; sub-plots). Precipitation inputs for 10 properties in Laikipia, Kenya were experimentally manipulated by increasing and decreasing total annual precipitation and the variability of intra-annual precipitation simultaneously; maximum viable livestock density was estimated by the Rangeland model as the density where the simulated livestock herd experienced one month of diet insufficiency out of a 24-month simulation. The range of PCI values inside the first quartile was 8.336–8.337; in the second quartile, 8.337–13.79; third quartile, 13.79–19.69; fourth quartile, 19.69–31.36.

Multiple regression analysis of the maximum viable livestock density under manipulated precipitation scenarios demonstrated that precipitation variability, in addition to total rainfall, has significant impacts on livestock production viability. As expected, total precipitation was a strong positive predictor of maximum viable density (estimate ± standard error: 0.035 ± 0.013; p < 0.0001). Both PCI (0.015 ± 0.008; p = 0.07) and site (0.0028 ± 0.023; p = 0.95) were non-significant as main effects. PCI interacted strongly with precipitation, however (−0.0011 ± 7.6 e-5; p < 0.0001), limiting the positive effect of precipitation on viable density when intra-annual variability was high (Fig. 3). The overall fit of the model including site and precipitation factors was good (adjusted R^2^ = 0.87).

### Land-sharing with livestock: what is the likely range of wildlife densities that may also be supported

**Figure 4:**
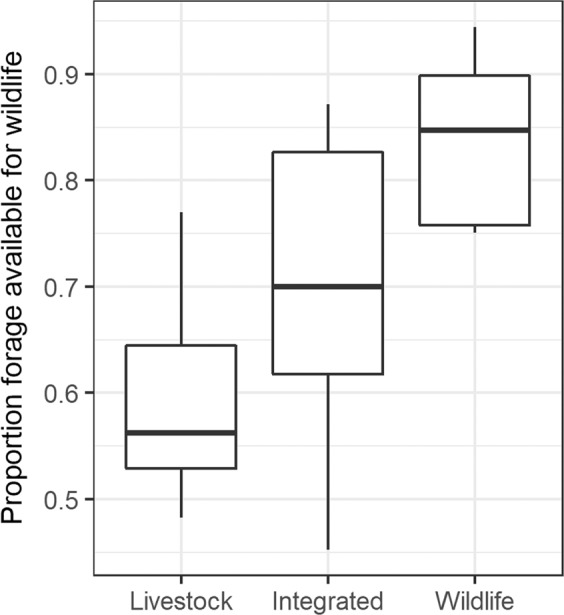
Estimated viable wild grazer density on properties managed predominately for livestock, wildlife, or livestock-wildlife integration, as proportion of maximum viable grazer density. Management strategies were derived from dung ratios; see Keesing *et al*.^17^ for management strategy classification methods.

Current estimated viable wild grazer densities, based on the difference between maximum viable density under long-term average precipitation and reported livestock densities, varied across the landscape from 0.23 to 1.17 cattle per ha, or 45–94% of the maximum utilization (Supplementary Table S2). Properties that had higher livestock-to-wildlife ratios based on dung surveys (*cf Keesing et al.*^17^) showed a higher utilization rate of the space by livestock–that is, reported livestock densities made up a larger percentage of the maximum viable density on those properties compared to other properties (Fig. 4). While cattle appear to consume more than twice the amount of available productive capacity on these livestock-dominated properties as compared to wildlife-oriented properties, at least half of the viable forage for grazers on livestock properties is still unutilized by cattle.

**Figure 5:**
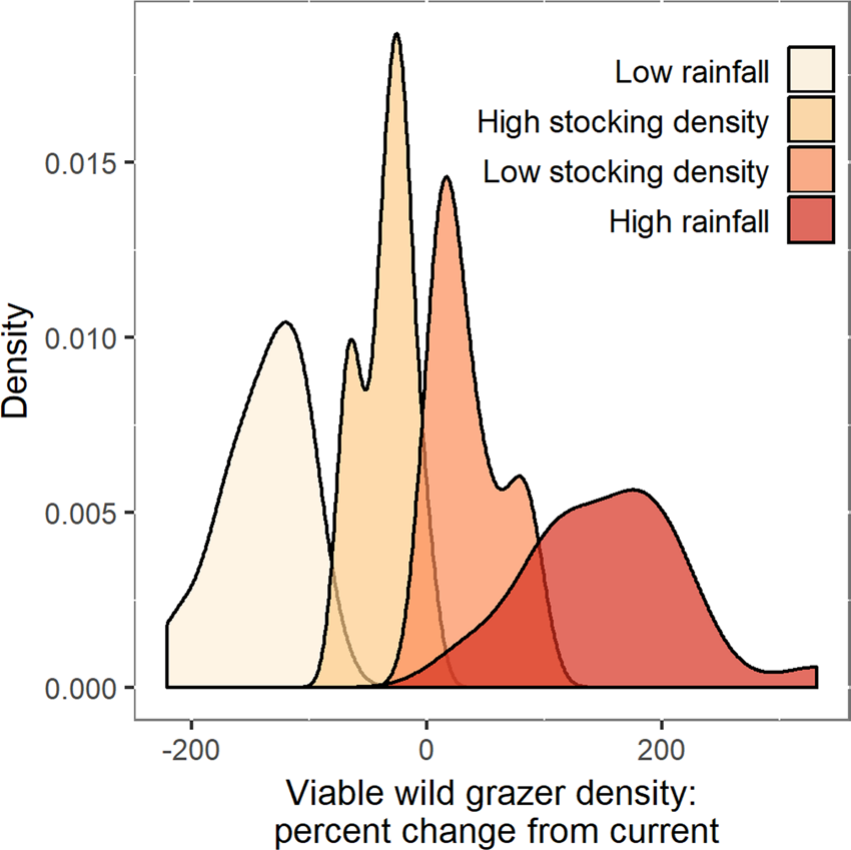
Density plot, percent change in viable wild grazer density after cattle offtake under extreme climate and management scenarios. Potential viable density of grazing wildlife under climate scenarios was calculated from current reported cattle stocking density and the maximum viable cattle density under decreased or increased rainfall; potential viable density of grazing wildlife under extreme management scenarios was calculated from maximum viable grazer density under current climate with region-wide switch to low or high cattle stocking density. Although analysis was conducted for 25 properties, only 22 properties reporting current stocking density are shown here.

Our scenario analysis showed that considering realistic ranges of yearly precipitation and management in the study region, precipitation impacted viable wild grazer density much more strongly than cattle stocking density (Fig. 5). Decreased precipitation such as seen in drought years resulted in a greater than two-fold reduction in viable wild grazer density on some properties, while viable wild grazer density was increased by up to 330% under increased precipitation. In contrast, increasing the cattle stocking density (applying the highest observed stocking density for the region across properties, with the largest differences appearing in the currently most lightly stocked properties) reduced the forage for wildlife by only up to 71%, and decreasing the cattle stocking density (applying the lowest reported stocking density across properties) increased forage for wildlife by up to 91%. Thus, a realistic range of total annual rainfall in the region drives 3.6 times larger differences in estimated viable wild grazer densities than the range of observed management extremes in the system.

## Discussion

We present here a new coupled model with lightweight data requirements that can be used to investigate management scenarios on rangelands that are typically out of reach in low-data environments or at the larger spatial extents of many conservation contexts. The Century model has been tested in its ability to predict grass growth around the world, including in response to herbivore pressure (e.g.^19–22^), yet has been limited in its applicability in rangelands because it is so difficult to know the density of herbivores in many systems *a priori*. By coupling Century with a basic herbivore physiology model, we are able to examine trends in forage availability and reliability across a heterogeneous landscape and under variable climate.

The *back-calculate management* routine proved to be a powerful way to accurately estimate grazing pressure given one biomass measurement. Using empirical biomass measurements to back-calculate management at OPC reduced the mean squared error of prediction (MSEP) by more than 1,188,000 kg/ha over simulations using preset grazing schedules from nearby regions. Indeed, calibrating the model in this way changed those starting assumptions by 31–230%, from removing all scheduled grazing events to adding 18 months of grazing events to the 2-year calibrated period, across locally heterogeneous sites. The small sample size of sites did not allow us to assess the uncertainty associated with this improvement in MSEP, for example through cross-validation^27^. Correlations with empirically-estimated cattle dung density, however, showed that the routine accurately differentiated between sites with different levels of grazing pressure. This is an important advance because grazing intensity impacts both within-season dynamics, such as phenology and forage nutritive quality, and longer-term effects via soil carbon and nutrient cycling^28^.

The approach described here lifts a critical data barrier in characterizing past site management. While information to describe recent grazing pressure is crucial to understanding and forecasting site dynamics, it is often proprietary and is typically available only at coarse administrative scales that may contain heterogeneous management practices. The method of estimating grazing intensity demonstrated here also suggests the possibility of using remote sensing of biomass to calibrate ecosystem models at landscape and larger scales (as previously shown for NDVI^29^). This more closely matches the scale of policies or incentives made by many decision-makers, and will enable prioritization of management interventions at that scale.

Our comparisons with empirical grazer densities support that the Rangeland model was able to meaningfully estimate recent past grazing pressure, but assessments of the individual submodels (described in Appendix S1) suggest that absolute values predicted by the model should be interpreted cautiously. Predictions by Century of the growth of ungrazed biomass were positively, but nonsignificantly, correlated with empirical grass growth (Fig. 1, Appendix S1), while the animal submodel showed a small tendency to underestimate forage intake (mean bias = 0.11 kg/day, or 3.2% of mean daily forage intake; Fig. 3, Appendix S1). Additionally, because each of the two linked models are associated with known sources of uncertainty^30,31^, linking them may potentially compound this uncertainty. The *back-calculate management* routine provides an estimate of past grazing history that may be impossible in many cases to obtain from other sources, but future users of the routine should remain aware that the full uncertainty associated with coupling Century with the ruminant submodel has not been fully quantified.

Our experimental measures of livestock viability under perturbed climate scenarios showed that intra-annual variability in precipitation matters nearly as much as the total amount, almost flattening the relationship between precipitation and livestock production viability in the highest-variability scenario. Understanding the role of variability in a changing climate will be increasingly important in semi-arid regions like Laikipia, where increasing rainfall variability has already been linked with productivity declines in agriculture over a recent 30-year period^32^. While regional- to global-scale studies using remotely sensed estimates of rangeland condition have suggested that precipitation variability is a major driver of condition, the relative impacts of animal stocking *vs*. climate remain contentious^33,34^.

The stocking densities where forage was insufficient to meet the simulated herd’s nutritional demands under average climate conditions (0.7 animals/ha and above) were much higher than most reported densities in the region, where the mean reported stocking density was only 0.25 animals/ha^17^. This apparent under-utilization of forage resources, which may partially be driven by the model’s tendency to underestimate livestock intake (Supplementary Appendix S1), likely also reflects a strategy of livestock managers in Laikipia to reduce vulnerability to temporal variation in forage availability. Common drivers of forage shortfalls include climate extremes (which were not reflected in the average climate that was used to drive simulations), and potentially incursions by livestock from neighboring areas. Because grazing incursions usually occur during periods of drought^35^, these stressors produce a compound effect. Some properties in the region provide supplemental feed for livestock in cases of extremely poor environmental conditions^36^, so the average conditions simulated here may not adequately describe the intake needs of livestock under drought.

Climate was also the primary driver of our simplified metric for wildlife habitat, describing the density of wild grazers that could be supported by forage after cattle offtake: our extreme climate and management scenarios suggest that within the bounds of observed conditions in the region, climatic conditions during any particular time period limit available resources for wildlife much more strongly than competition for forage with livestock. Historical evidence supports that grazing wildlife and livestock are more likely to compete during periods of drought^37^ and that human-wildlife conflict is exacerbated by environmental stress^38^. Increasingly severe drought cycles are considered one of the major drivers of ongoing declines in wildlife populations in East Africa^2^. Although current forage resources in Laikipia are sufficient to support co-management of domestic livestock with wildlife, increasingly frequent and extreme drought conditions may pose the greatest impediment in the future to livestock-wildlife integration. Conversely, these results also suggest that the more mesic, productive properties in the region may have ample forage resources to support additional grazers, and should therefore see an opportunity to reap potential ecological and economic benefits of integrating domestic livestock with wildlife^39^.

Because the Rangeland model is point-based, predicting forage growth and consumption by herbivores at a single location, it is limited in its ability to model spatial interactions between livestock and wildlife. The model cannot in its present form, for example, account for facilitation of highly nutritious forage for wildlife following abandonment of cattle bomas^40^. Nor can the model represent spatiotemporal niche partitioning between livestock and wildlife that may reduce competition within large and heterogeneous properties^41^, or potential impacts of domestic livestock and their herders on the distribution of real or perceived predation risk for wildlife^42^. Spatial interactions are an important dimension of livestock-wildlife coexistence in the Kenyan landscape in particular^43^, where habitat selection by wildlife is often related to current or recent presence of grazing livestock^41^. However, in regions like Laikipia where fragmentation of the landscape increasingly limits the ability of wildlife to move adaptively in response to large-scale resource heterogeneity, including heterogeneity created by livestock, competitive interactions with livestock may dominate^44^. The possibility of extending the Rangeland model to a gridded, rather than point-based, implementation is a promising direction for the future; this extension of the model would allow for more explicit consideration of the role of animal movement and spatial segregation in livestock-wildlife coexistence.

The Rangeland model is also restricted to representing herbaceous biomass only, and does not currently predict the growth or consumption of shrubs or trees. The model is therefore unsuitable for simulation of browsing or mixed-feeder herbivores, which constitute a large portion of wildlife in Laikipia^45^. Further, because encroachment of woody plants into grassy habitats may limit forage availability for cattle^46^, the potential role of browsing wildlife in suppressing woody encroachment^40^ is another important dimension of interactions between livestock and wildlife that is not addressed by the model.

The constraint that the model simulates only herbaceous vegetation also limits its ability to represent all domestic livestock production in Laikipia. On a few of the properties surveyed in Laikipia, sheep and goats (identified collectively as “shoats” because their dung could not be reliably differentiated) represented a majority of livestock dung, although the median value was only 36%^17^. While parameters to apply the ruminant submodel to sheep are readily available from GRAZPLAN^19^, it would be incorrect to apply the model in its current form to predict the viability of goat production in areas where woody vegetation provides a significant source of forage. This is an important consideration, particularly as the abundance of shoats in Laikipia is increasing^47^.

The application of the Rangeland model described here assumes relatively steady-state patterns of productivity across properties within Laikipia, driven by our modeling choice to use average annual precipitation as model inputs on regional properties. Rather than interpreting the metric of maximum viable density of livestock as carrying capacity, which would presume that an equilibrium was established, however, our simulations using experimentally manipulated precipitation scenarios bridge the model conceptually with non-equilibrium theory^5,48^. The rainfall manipulation experiment showed that high intra-annual variability of precipitation limited the viable grazer density in addition to total rainfall (Fig. 3), and showed that the maximum viable density metric reflected the constraints of a particular precipitation regime, not a stable site characteristic. A potential future direction for model development would be to allow herbivore densities to fluctuate over time; this would extend the model’s usefulness in non-equilibrial systems^49^. Given that Century does not simulate changes in plant community composition, the Rangeland model is not applicable on its own to simulate grazing-induced transitions between multiple steady states^50^, though it could evaluate differences between those states if coupled with a state and transition model.

This application of the Rangeland model in Laikipia focuses on the primary model outputs of forage availability for grazing livestock and wildlife. Given the range of ecosystem-level quantities simulated by Century, however, the model clearly has potential to be useful in many other contexts. For example, Century’s detailed representation of soil carbon suggests that the model can be useful in predicting grazing dynamics in grasslands recently converted from forest or other vegetation types^51^. There is great potential to model process-based linkages between viable livestock production and other ecosystem processes that can be modeled with Century, including carbon and nutrient cycling, which could then be used as drivers to link grazing dynamics with other ecosystem services models. By linking Century with a ruminant physiology model, it is also likely that the model could address greenhouse gas emissions from livestock in a manner that is fully integrated with other ecosystem services. Because the characteristics of ruminant metabolism driving methane emissions from livestock are highly sensitive to forage protein content and digestibility^52^, we caution that any application of the Rangeland model addressing greenhouse gas emissions must carefully validate the plant parameterization controlling these characteristics.

The challenge as well as the importance of understanding temporal variability and spatial heterogeneity grows as we work across larger scales – and landscape or regional level information is what most conservation decisions demand. Any form of land management in grasslands will often have to contend with livestock operations, and understanding the impact of grazing in concert with climate variability across large and spatially heterogeneous regions requires a new approach to rangeland modeling. The model described here, which can help to tease apart the interaction between total precipitation, precipitation variability, and grazing intensity, across space and time, can help to better forecast rangeland dynamics under simultaneous pressures from changing climate and management. By integrating Century’s ecosystem flows with a basic ruminant diet selection and physiology submodel, the Rangeland model extends the capability of Century beyond its historical applications, adding dynamic diet selection and productivity of livestock to the purview of Century’s whole-ecosystem approach. This new modeling approach provides an opportunity to include the impacts of grazing on rangelands and the benefits we derive from these systems, across heterogeneous landscapes and for a wide variety of decision contexts.

## Supplementary information

Supplemental Information

## Data Availability

All data supporting results in the manuscript are archived in the public data repository Mendeley Data (10.17632/4m3xybvdb6.2). The software used to generate the results in the manuscript is publicly available at a permanent code archive (10.5281/zenodo.1557484). The Rangeland Production model described in this study is free, open-source and available for download from the archive. Full installation instructions and inputs used to run the model are also available at the archive link above. The model currently requires that users obtain a copy of the Century model from its developers.
