# Supplementary material for: A coupled forage-grazer model predicts viability of livestock production and wildlife habitat at the regional scale
Source: Sci Rep. 2019 Dec 30;9:19957. doi: 10.1038/s41598-019-56470-3 (PMC6937286; doi:10.1038/s41598-019-56470-3)
Supplement: Supplementary file 1 — Supplemental Information [file 41598_2019_56470_MOESM1_ESM.pdf]

# A coupled forage-grazer model predicts viability of livestock production and wildlife habitat at the regional scale

Virginia A. Kowal, Sharon M. Jones, Felicia Keesing, Brian F. Allan, Jennifer M. Schieltz, Rebecca Chaplin-Kramer

## Supplemental information

**Figure S1.** Map of the study area, showing average annual rainfall across Laikipia County. Empirical measurements were taken on 25 properties across and immediately adjacent to the county; to protect privacy, exact property locations are not shown. Inset at left shows the location of Laikipia County in Kenya. Detail inset shows Ol Pejeta Conservancy (OPC), a property where more detailed empirical data were collected. Numbered triangles on OPC show weather stations, while vegetation/dung transect locations appear as filled dots. Grey boxes around each weather station show the 1600 ha area within which empirical biomass and livestock density were aggregated for that site. Only weather stations with non-overlapping 1600 ha areas were included in empirical comparisons with model results, and hence only those weather stations are shown here.

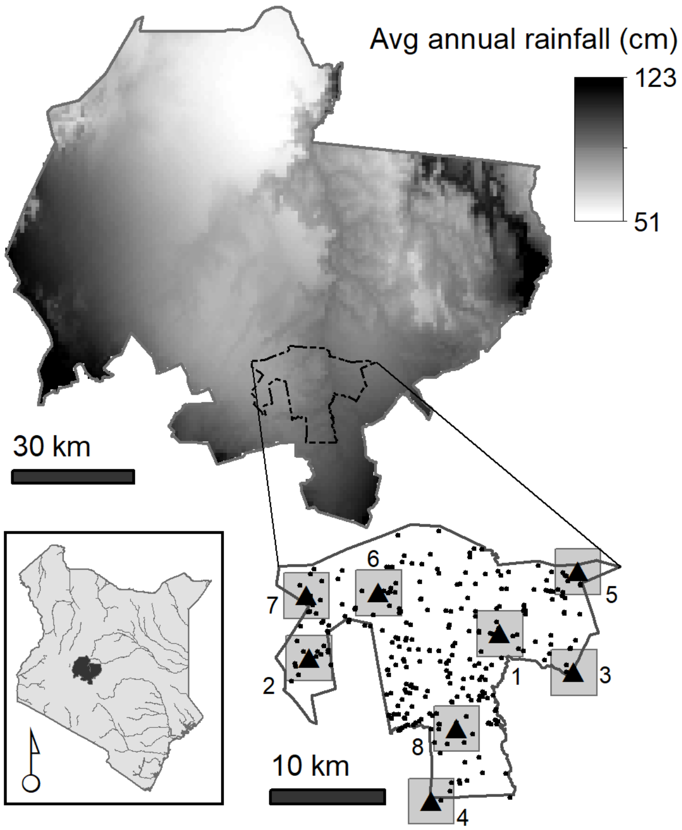

**Figure S2.** Comparison of biomass from simulated (triangle) and empirical (circle) sources on the date of empirical biomass measurement. The panel on the left shows simulated biomass at the target match date without calibration of the management history by the *back-calculate management* routine; on the right is simulated biomass when simulated management history at each site was estimated by the *back-calculate management* routine. X axis corresponds to site labels in Fig. S1.

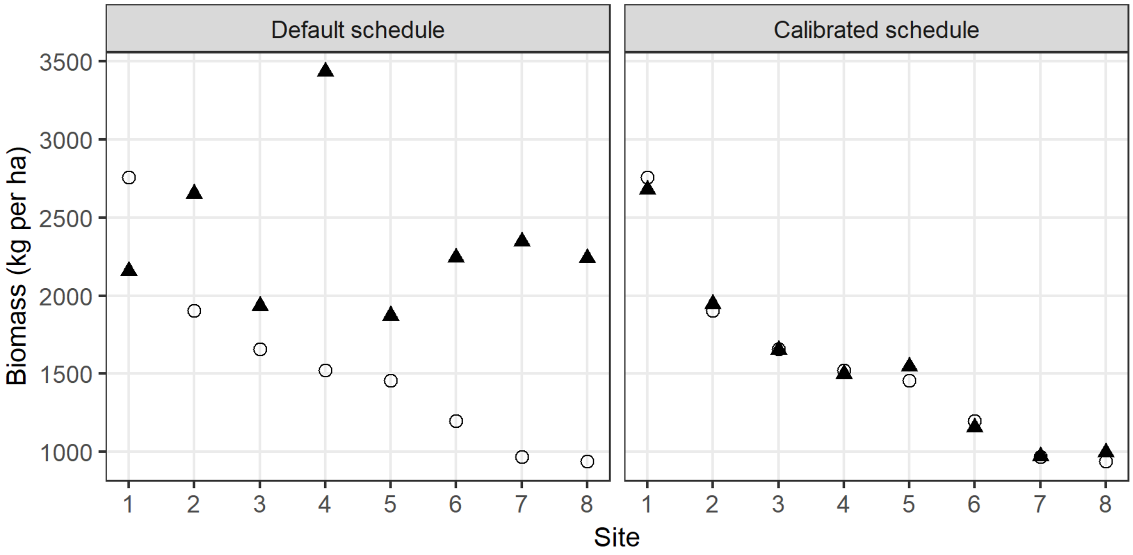


**Figure S3.** Number of months where the herd's energy requirements were not met, in relation to average annual rainfall at each property. Sub-plots show three stocking density levels (columns; 0.7 - 1.33 animals/ha) and six tested months of conception relative to the model starting month (rows; from 12 months prior to starting month to 8 months after model starting month). This figure illustrates that while precipitation and stocking density are strong drivers of livestock viability as defined by diet sufficiency, conception month does not strongly predict livestock production viability.

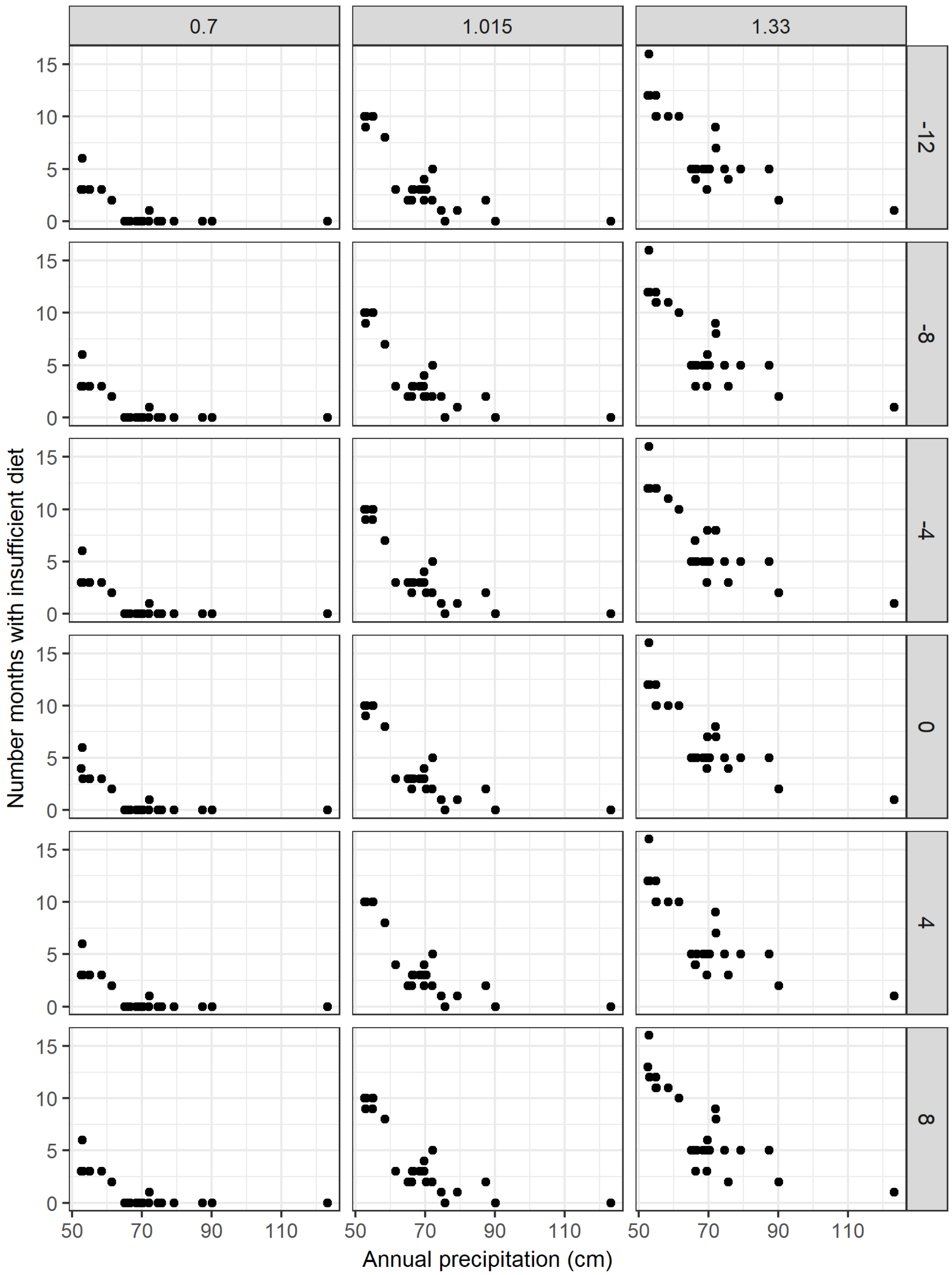

0.7
1.015
1.33
-12
-8
-4
0
4
8
15
10
5
0
50
70
90
110
Number months with insufficient diet
Annual precipitation (cm)


40

**Table S1.** Summary of correlations between modeled grazing intensity, as calculated by the *back-calculate management* routine, and dung density of animal functional groups. Statistically significant correlations indicated by *.

| Functional group | Correlation | p value |
|---|---|---|
| Cattle and buffalo | 0.79* | 0.02 |
| Other grazer | -0.132 | 0.76 |
| Carnivore | -0.53 | 0.18 |
| Mixed | 0.06 | 0.88 |
| Browser | 0.03 | 0.95 |

**Table S2.** Derivation of viable wild grazer density, the density of grazing wildlife that could be supported after cattle forage offtake according to maximum viable cattle density and reported cattle stocking density. Average annual rainfall was calculated from Worldclim v 1.4 [1], giving average rainfall during the period 1960-1990 at the property centroid. Properties marked “NA” did not report stocking density. Management strategies dominated by livestock, wildlife, or integration of livestock with wildlife were classified by [2] from the density of dung piles. Properties were anonymized following [2].

| Property | Ecological classification | Average annual rainfall (cm) | Maximum viable density (cattle/ha) | Stocking density (cattle/ha) | Viable wild grazer density (cattle/ha) |
|---|---|---|---|---|---|
| A | Integrated | 66.2 | 1.00 | 0.13 | 0.87 |
| B | Wildlife | 90.2 | 1.32 | 0.20 | 1.12 |
| C | Livestock | 69.8 | 0.89 | 0.42 | 0.47 |
| D | Integrated | 66.6 | 0.83 | 0.16 | 0.68 |
| E | Integrated | 52.6 | 0.56 | 0.22 | 0.34 |
| F | Livestock | 69.5 | 0.93 | 0.43 | 0.50 |

| | | | | | |
|---|---|---|---|---|---|
| G | Livestock | 72.2 | 0.71 | 0.37 | 0.34 |
| H | Wildlife | 123.3 | 1.24 | 0.07 | 1.17 |
| I | Integrated | 52.9 | 0.56 | 0.17 | 0.39 |
| J | Livestock | 65.1 | 0.86 | 0.29 | 0.57 |
| K | Livestock | 72.1 | 0.86 | NA | NA |
| L | Livestock | 74.7 | 1.05 | 0.24 | 0.81 |
| M | Integrated | 70.3 | 0.93 | 0.13 | 0.79 |
| N | Wildlife | 55 | 0.55 | 0.14 | 0.41 |
| O | Wildlife | 69.7 | 0.93 | 0.09 | 0.83 |
| P | Wildlife | 79.3 | 1.05 | NA | NA |
| Q | Integrated | 61.5 | 0.68 | 0.35 | 0.33 |
| R | Integrated | 75.7 | 1.20 | 0.19 | 1.00 |
| S | Livestock | 55.1 | 0.55 | NA | NA |
| T | Wildlife | 68.5 | 0.86 | 0.21 | 0.65 |
| U | Integrated | 58.5 | 0.62 | 0.16 | 0.46 |
| V | Integrated | 54.9 | 0.55 | 0.18 | 0.37 |
| W | Integrated | 53.1 | 0.50 | 0.27 | 0.23 |
| X | Integrated | 87.4 | 0.90 | 0.34 | 0.56 |
| Y | Livestock | 66.4 | 0.82 | 0.34 | 0.48 |

## References


1. Hijmans, R.J., Cameron, S.E., Parra, J.L., Jones, P.G. & Jarvis, A. Very high resolution interpolated climate surfaces for global land areas. *Int. J. Climatol.*, **25,** 1965–1978 (2005).

2. Keesing, F., *et al.* Consequences of integrating livestock and wildlife in an African savanna. *Nat. Sustainability*, **1,** 566-573 (2018).

## Appendix S1: Supplemental Methods for Case Study Application

### Field data collection

Empirical data describing standing biomass and wildlife dung were collected at 286 100 m transects on Ol Pejeta Conservancy (OPC), a large (37,000 ha) property in southern Laikipia (Fig. S1). Annual surveys of vegetation and dung along each transect were conducted in July-August 2014 and 2015 (cf. methods in [1]). Transects were located semi-randomly on the conservancy across a gradient of cattle activity as described in [2].

Biomass was estimated at each transect with a pasture disk meter (PDM; [3]) which was dropped once every 10 m along the transect to estimate vegetation height. PDM measurements were transformed to dry weight biomass with a calibration coefficient estimated by regression (Ol Pejeta Conservancy Ecological Monitoring Department, personal communication), and averaged within transect to give a single biomass estimate per transect per date of sampling.

Simultaneous with biomass measurement, trees, shrubs and animal dung were counted if they fell within 0.5 meter of the transect center line. When possible, animal dung was identified to species; the dung of cattle (domestic cattle) and African buffalo (*Syncerus caffer*) could not be reliably distinguished in the field and so their density was combined into a single class. Because buffalo populations in Laikipia are approximately 1-2% the size of cattle populations [4], we consider this combined buffalo and cattle class to predominantly reflect cattle.

### Model inputs and parameterization

Soil inputs for all model simulations were derived from SoilGrids 250 m soil maps for Africa [5]. Century parameters were taken from the grass parameterization described by [6] for tropical C4 grass in Nairobi National Park. For the spin-up period, covering time prior to the collection of empirical weather data, we used average climatic inputs calculated from the historical data for the site.

Each simulation was driven by monthly temperature and precipitation data collected at that site. For OPC simulations, monthly temperature and precipitation data were aggregated from daily records that have been collected at weather stations across OPC since the late 1990s (Fig. S1). For the regional simulations, temperature and precipitation inputs were derived at a 1

km scale from Worldclim v. 1.4 current conditions at the property centroid, describing average monthly climate from approximately 1960 to 1990 [7].

The composition of the livestock herd (i.e., relative proportions of each age and sex class) was taken from herd census records collected on OPC in 2015 (OPC livestock manager, personal communication). The average weight of each age/sex class was also supplied by OPC; we calculated the age for each class according to the Rangeland model such that the animals were assumed to be in median body condition for their age and reproductive status.

Reproductive phase inputs to the Rangeland model were informed by a literature search. Calving interval, giving the total length of the reproductive cycle, is strongly influenced by cow body condition and is highly variable in Kenya [8]. We used a calving interval of 15 months, which is intermediate among reported values [8], [9], [10]. Conception month, an input that must be supplied relative to the modeled time period, determines how periods of high energy demand by the herd (e.g. peak of lactation) coincide with periods of high or low rainfall and productivity. Most Kenyan herders do not encourage coordinated calving among their cows, and conception and calving follow only a weakly seasonal pattern [11], [12]. Because we expect diet sufficiency to be highly sensitive to this uncertain input, we ran the model using a range of conception months to assess the sensitivity of model results.

**Full methods: back-calculating management**

We used the *back-calculate management* routine to estimate grazing intensity by matching the final biomass measurement taken at each weather station on Ol Pejeta Conservancy. We used 100 kg/ha as the target threshold, meaning that the routine concluded successfully when the difference between empirical and simulated biomass at the empirical measurement date was less than 100 kg/ha. We allowed the routine to run for a maximum of 40 iterations, calibrating the grazing schedule and intensity for a maximum of 24 months prior to the empirical measurement date.

The starting schedule for the *back-calculate management* routine was the historical grazing regime described by [6] for Nairobi National Park, which included removal of 12% of standing live biomass and 6% of standing dead biomass in April, May, June, July, October and November of each year. Like [6], we also included a fire event every 5 years in the month of

March. This fire event was assumed to remove 85% of standing live biomass, standing dead biomass, and litter. This management schedule was assumed to be in place for a spin-up period of 5000 years prior to the empirical measurement date.

For comparison to model outputs, we summarized biomass and animal density observations across transects by proximity to weather stations on OPC, as shown in Figure S1. To derive empirical measurements from field data for comparison with model outputs, we aggregated biomass and animal density estimates collected at transects within a 1600 ha area surrounding each site (i.e., 2 km in the x and y directions; grey boxes in Fig. S1). We restricted empirical biomass estimates to include only those transects with low recorded shrub and tree cover (<6 trees and <8 shrubs counted along the 100 m transect, thereby excluding the top 20th percentile of trees and shrubs across transects) to match our modeling focus on grass. Because vegetation and dung transects were conducted multiple times across OPC during 2014 and 2015 but with semi-random temporal and spatial distribution (cf. methods in [2]), each site was associated with a different number and temporal spacing of vegetation and dung measurements.

**Validation of sub-models**

As a widely applied ecosystem model originally developed in grasslands, Century has been extensively tested and validated in diverse rangeland environments (e.g., [6], [13], [14], [15]). In addition, we have strong confidence in the predictions of Century for the current application because the model was not modified, and because we relied on an existing parameterization validated by [6]. The diet selection and energy requirement routines of the ruminant physiology submodels have also been extensively validated as part of the GRAZPLAN model ([16] and references therein).

However, to ensure reliability of each submodel, we verified our application of the models in Laikipia with additional validation tests of model components, which supported the many published validations already conducted for each submodel.

**Validating Century: comparison to caged plots**

We validated the ability of Century to predict grass growth in Laikipia with comparison to the growth of grass measured at 33 grazing exclosure sites on Ol Pejeta Conservancy (OPC) in

2012, 2013, and 2014. Biomass in the exclosure sites was sampled with a calibrated pasture disk meter once every three weeks over a twelve-week period beginning in June 2012, June 2013, and January 2014. Following the first empirical biomass measurement, a 1m by 1m exclosure cage made of rebar and chicken wire was installed, which prevented grazing of the grass inside. The twelve-week time series of empirical measurements collected at each site therefore describes the growth of previously grazed grass without grazing. The sites were located in managed livestock pastures that have been grazed by livestock for many years, but the exact timing and intensity of livestock use was unknown (OPC Ecological Monitoring Department, personal communication). Rainfall on OPC during the period of field data collection was approximately average in 2012 and 2013, and slightly below average in 2014, according to daily precipitation records collected at weather stations on the conservancy.

We used the *back-calculate management* routine to match initial biomass in each plot and compared simulated biomass growth predicted by Century to empirical biomass growth for those plots where the *back-calculate management* routine successfully matched initial biomass. Because empirical biomass was collected at three-weekly intervals, while Century reports biomass at monthly intervals, we estimated simulated biomass at empirical measurement dates through linear interpolation. We assessed the fit of simulated time series to the empirical biomass time series by calculating the pearson correlation between first differences (i.e., differences between consecutive observations) of each series. We assessed the relationship between model performance and average precipitation at the simulated site by calculating the difference between observed and predicted biomass growth at each sampling date, and comparing the distribution of these differences among levels of annual average precipitation calculated from weather records.

Climate inputs were derived from the closest OPC weather station to each site; the 33 sites where data were collected were associated in this way with four weather stations on OPC. Other model inputs were derived from SoilGrids 250 m soil maps for Africa [5] and a grass parameterization for Century derived by [6] for tropical C4 grass in Nairobi National Park.

The *back-calculate management* routine successfully matched empirical management for 27 of the 33 sites tested. Five of the six sites that were not successfully matched had empirical biomass that was higher than what was possible to produce given simulated conditions, even

when grazing was removed from the site schedules for up to two years preceding the empirical measurement date.

Of the sites where the *back-calculate management* routine succeeded, the majority (63% of sites) required that grazing pressure be increased from the beginning template historical management schedule; 22% required that grazing pressure be reduced; and four sites (15%) required no modification to the beginning grazing schedule to match empirical biomass. Out of the 17 sites where grazing pressure was increased to match empirical biomass, 13 sites matched the empirical measurement only with grazing scheduled continuously through the two years prior to the empirical measurement date. These sites showed removal of up to 60% of live biomass each month by herbivores.

The agreement between modeled and empirical biomass growth in the caged plots was weak: the correlation between first differences of simulated and empirical time series was positive, but not significant (pearson $\rho = 0.15$; $p = 0.14$). Despite this, Century did not consistently over- or under-predict grass growth, and the time series of simulated and empirical biomass appeared similar for most sites (Fig. 1).

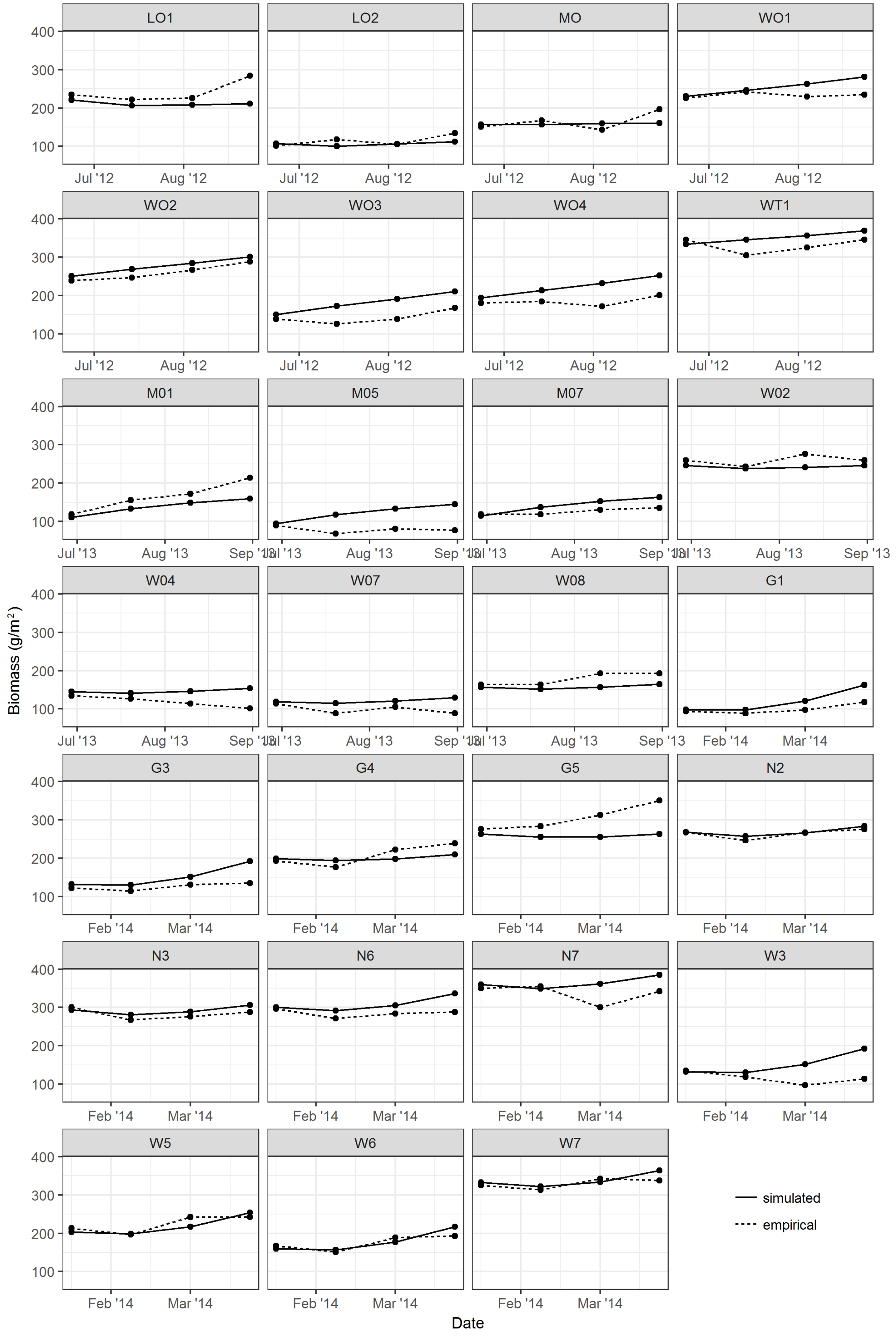
LO1
LO2
MO
WO1
WO2
WO3
WO4
WT1
M01
M05
M07
W02
W04
W07
W08
G1
G3
G4
G5
N2
N3
N6
N7
W3
W5
W6
W7
Jul '12
Aug '12
Jul '13
Aug '13
Sep '13
Feb '14
Mar '14
100
200
300
400
Biomass (g/m²)
Date
simulated
empirical

Figure 1. Simulated and empirical biomass for twenty-seven caged sites in Laikipia, Kenya. Simulated values on three-weekly empirical measurement dates were obtained from monthly simulated time series via linear interpolation. The *back-calculate management* routine was used to calibrate grazing intensity prior to the first biomass measurement shown here.

Average annual precipitation at the four weather stations driving simulations ranged from 62.7 cm to 71.8 cm; the difference between observed and predicted biomass growth at each sampling date did not discernibly differ according to annual precipitation (Fig. 2). The distribution of differences between observed and predicted biomass by average precipitation suggested that the model tended to slightly overpredict change in biomass in drier environments (i.e., the mean of differences at the lowest-rainfall site was below zero), while the opposite was true at wetter sites (the mean of differences at higher rainfall sites was above zero, indicating that the model under-predicted biomass growth).

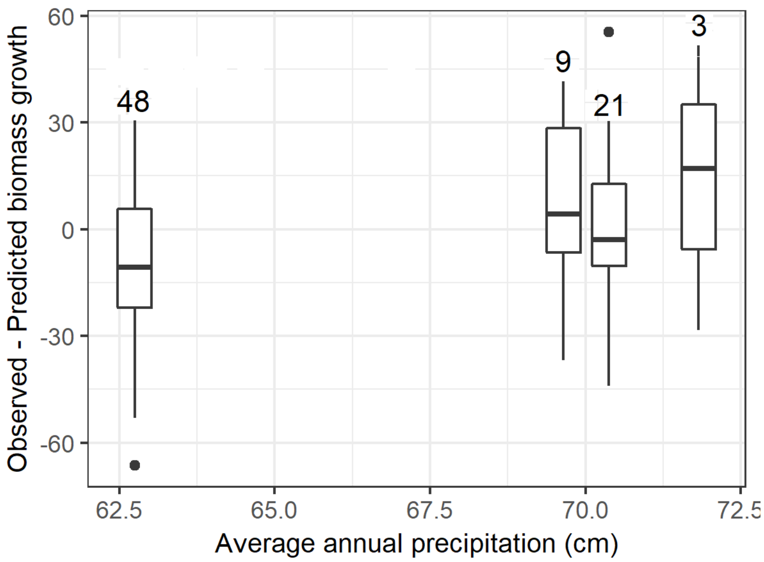


Figure 2. Relationship between model performance, as quantified by the difference between simulated and observed change in biomass, and average annual precipitation at the weather station closest to the empirical sampling site. Labels above each box-and-whisker give the sample size, which is the number of biomass measurements taken at the sites driven by that weather station.

**Validating the ruminant physiology submodel: comparison to published feeding trials**

The ruminant physiology submodel was adapted from the GRAZPLAN model, which itself is based on the Australian feeding standard [17]. Due to the large size and diverse climatic conditions of the Australian continent, the national feed guidance for Australia necessarily includes guidance for both temperate and tropical systems. The GRAZPLAN model therefore includes published parameter sets that were derived for both cool- and warm-season grasses, and for cattle of *Bos taurus* (temperate) and *B. indicus* (tropical) breeds [18]. The majority of published validations of GRAZPLAN, however, apply to temperate systems [19], [20]. To validate our application of the adapted ruminant submodel to the tropical grasslands of Kenya, we focused our test of the submodel on tropical feeds and cattle.

We used empirical intake published by [21] to test the ability of our basic ruminant physiology submodel to predict intake from forage types differing in nutritive value. This study, conducted in Tanzania, measured intake and daily gain achieved by Ayrshire-cross bulls fed with 18 different forage and feed types (Table 1). The bulls had an average age of 456 days and average weight of 163 kg. The standard reference weight (SRW) for Ayrshire-cross animals is uncertain, depending on the percent indigenous breed [22]. We therefore tested a wide range of SRW values, from a minimum of 224 kg for bulls (enforcing median body condition for the animals' weight and age) to 840 kg for bulls (corresponding to 600 kg for a mature female, greater than the typical size of Kenyan Ayrshire cows [23]. In the publication, all forage types were supplied *ad libitum;* we recreated this level of availability in the simulation by assuming constant biomass availability of 400 kg/ha.

We replicated the supplementation reported in the study: 1 kg cotton seed cake per day per animal. Nutritive values for the supplement were not given by [21]; we assumed a dry matter digestibility of 0.8 [24] and crude protein content of 338.1 g/kg [25].

Table 1. Quality characteristics of 18 feed types included in the study published by [21]. CP: crude protein, DMD: dry matter digestibility.

| Feed type | CP (g/kg) | DMD (g/kg) |
|---|---|---|
| Green Kilima | 73 | 649 |
| Dry Kilima | 41 | 549 |
| 30 g/kg urea on Kilima | 79 | 587 |
| 50 g/kg urea-treated Kilima | 81 | 645 |
| Green Malawi | 88 | 686 |
| Dry Malawi | 49 | 59 |
| 30 g/kg urea on Malawi | 89 | 604 |
| 50 g/kg urea-treated Malawi | 98 | 678 |
| Maize stover tops | 43 | 615 |
| Guatemala grass | 109 | 638 |
| Setaria grass | 9 | 685 |
| Napier grass | 114 | 643 |
| Canadian wonder straw | 66 | 651 |
| Belabela bean straw | 48 | 583 |
| Rhodes grass (hay) | 44 | 612 |
| Rhodes grass (green) | 67 | 642 |
| Banana leaves | 127 | 507 |
| Banana pseudostems | 38 | 768 |

We replicated the published experiment by running the simulation for 60 days, recording predicted intake of intake of forage at each daily step. We evaluated the model's ability to match published values for average daily intake of each feed type with simple linear correlation. We calculated mean bias of predicted intake at each SRW value in order to evaluate whether the model tended to under- or over-predict intake (equation (1); [26]).

$$MB = \frac{\sum_{i=1}^{n} \left(O_i - P_i\right)}{n} \tag{1}$$

where $O_i$ and $P_i$ are the observed and predicted intake, in kg/day, for the *i*th feed type, and *n* is the total number of feed types.

Simulated intake values were significantly correlated with empirical values reported by [21] across all tested SRW values, but correlations were strongest at low SRW, where animals were estimated by the model to be at median condition for their age and weight (Table 2).

Table 2. Pearson correlations of daily intake simulated by the beta Rangeland Production model and measured empirically by [21]. SRW, standard reference weight of bulls.

| **SRW (kg)** | **Relative condition** | **Correlation coefficient** | **p value** |
|---|---|---|---|
| 224 | 1.00 | 0.74 | < 0.001 |
| 301 | 0.89 | 0.69 | 0.002 |
| 378 | 0.81 | 0.63 | 0.005 |
| 455 | 0.75 | 0.59 | 0.01 |
| 532 | 0.70 | 0.56 | 0.02 |
| 609 | 0.65 | 0.53 | 0.02 |
| 686 | 0.61 | 0.51 | 0.03 |
| 763 | 0.58 | 0.49 | 0.04 |
| 840 | 0.55 | 0.48 | 0.04 |

Mean bias was negative at all but the smallest SRW value, showing that the model overestimated intake at the larger SRW values. The absolute value of mean bias was minimized at SRW=224, where the model slightly underestimated intake across feed types (mean bias = 0.11 kg/day; Fig. 3).

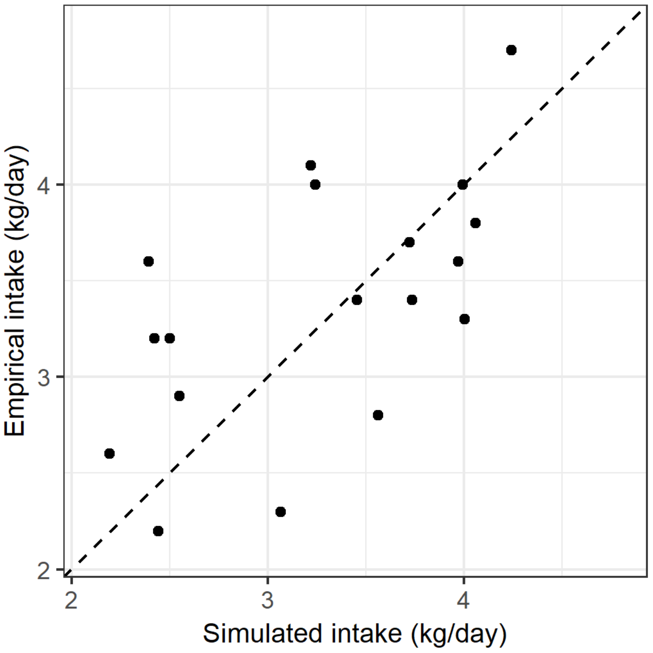


Figure 3. Simulated vs. empirical daily intake by bulls fed eighteen forage types differing in digestibility and crude protein content. Empirical values reported by [21]. Dotted line indicates 1:1 relationship. This plot shows values obtained at the standard reference weight (SRW) for bulls that maximized the simple correlation between observed and predicted intake, and minimized mean bias of prediction (SRW=224 kg).

Because the true SRW value for the bulls in the published study was unknown, it is reasonable to assume that the correct value was the one where the animals' weight would have indicated median body condition (i.e., SRW=224 kg). At this SRW value, the correlation between predicted and observed intake of the different feed types was high ($\rho = 0.74$; $p < 0.001$) and mean bias was low relative to the magnitude of predicted and observed values (mean bias = 0.11 kg/day, or 3.2% of mean daily intake across feed types). The tendency of the model to over-predict intake at higher SRW values, where the animals' weight would have implied low body condition, likely reflects the purposeful representation by the ruminant physiology model of increased compensatory intake at low body scores [18].

**References**

1. Keesing, F., *et al.* (2018). Consequences of integrating livestock and wildlife in an African savanna. *Nat. Sustainability*, 1, 566.

2. Schieltz, J.M., Okanga, S., Allan, B.F. & Rubenstein, D.I. (2017). GPS tracking cattle as a monitoring tool for conservation and management. *Afr. J. Range For. Sci.*, 34, 173–177.

3. Dörgeloh, W.G. (2002). Calibrating a disc pasture meter to estimate above-ground standing biomass in Mixed Bushveld, South Africa. *Afr. J. Ecol.*, 40, 100–102.

4. Kinnaird, M., O'Brien, T. & Ojwang, G. (2012). Sample count aerial surveys as a monitoring tool for wildlife and livestock: A case study from Laikipia County. In: *Report submitted to Laikipia Wildlife Forum, Nanyuki, Kenya*.

5. Hengl, T., *et al.* (2017). SoilGrids250m: Global gridded soil information based on machine learning. *PLoS One*, 12, e0169748.

6. Parton, W.J., *et al.* (1993). Observations and modeling of biomass and soil organic matter dynamics for the grassland biome worldwide. *Global Biogeochem. Cycles*, 7, 785–809.

7. Hijmans, R.J., Cameron, S.E., Parra, J.L., Jones, P.G. & Jarvis, A. (2005). Very high resolution interpolated climate surfaces for global land areas. *Int. J. Climatol.*, 25, 1965–1978.

8. Odima, P.A., McDermott, J.J. & Mutiga, E.R. (1994). Reproductive performance of dairy cows on smallholder dairy farms in Kiambu district, Kenya: Design, methodology and development considerations. *Kenya Vet.*

9. Ojango, J.M. & Pollott, G.E. (2001). Genetics of milk yield and fertility traits in Holstein-Friesian cattle on large-scale Kenyan farms. *J. Anim. Sci.*, 79, 1742–1750.

10. Ilatsia, E.D., Muasya, T.K., Muhuyi, W.B. & Kahi, A.K. (2007). Genetic and phenotypic parameters and annual trends for milk production and fertility traits of the Sahiwal cattle in semi arid Kenya. *Trop. Anim. Health and Prod.*, 39, 37–48.

11. Odhuba, E.K. (1988). The role of body weight changes and other factors in the control of fertility of beef cattle at Athi River Ranch, Kenya. *African Forage Plant Genetic Resources, Evaluation of Forage Germplasm and Extensive Livestock Production Systems*, 331.

12. Bekure, S., De Leeuw, P.N., Grandin, B.E. & Neate, P.J.H. (Eds.). (1991). *Maasai herding: analysis of the livestock production system of Maasai Pastoralists in eastern Kajiado District, Kenya*. ILCA Systems Study. ILCA (International Livestock Centre for Africa), Addis Ababa, Ethiopia.

13. Holland, E.A., Parton, W.J., Detling, J.K. & Coppock, D.L. (1992). Physiological responses of plant populations to herbivory and their consequences for ecosystem nutrient flow. *Am. Nat.*, 140, 685–706.

14. Schimel, D.S., e*t al.* (1994). Climatic, edaphic, and biotic controls over storage and turnover of carbon in soils. *Global Biogeochem. Cycles*, 8, 279–293.

15. Seastedt, T.R., Coxwell, C.C., Ojima, D.S. & Parton, W.J. (1994). Controls of plant and soil carbon in a semihumid temperate grassland. *Ecol. Appl.*, 4, 344–353.

16. Bryant, J.R. & Snow, V.O. (2008). Modelling pastoral farm agro-ecosystems: A review. *N. Z. J. Agric. Res.*, 51, 349–363.

17. CSIRO. (2007). Feeding Standards for Australian Livestock: Ruminants. 23. CSIRO Publishing.

18. Freer, M., Moore, A.D. & Donnelly, J.R. (2012). The GRAZPLAN animal biology model for sheep and cattle and the GrazFeed decision support tool. CSIRO Plant Industry, Canberra, ACT Australia.

19. Donnelly, J.R., *et al.* (2002). Evolution of the GRAZPLAN decision support tools and adoption by the grazing industry in temperate Australia. *Agric. Syst.*, 74, 115–139.

20. Cohen, R. D. H., J. P. Stevens, A. D. Moore, and J. R. Donnelly. (2003). Validating and Using the GrassGro Decision Support Tool for a Mixed Grass/Alfalfa Pasture in Western Canada. *Can. J. Agric. Sci.,* 83, 171–82.

21. Shem, M.N., Ørskov, E.R. & Kimambo, A.E. (1995). Prediction of voluntary dry-matter intake, digestible dry-matter intake and growth rate of cattle from the degradation characteristics of tropical foods. *Anim. Sci.*, 60, 65–74.

22. Lukuyu, M.N., Gibson, J.P., Savage, D.B., Duncan, A.J., Mujibi, F.D.N. & Okeyo, A.M. (2016). Use of body linear measurements to estimate liveweight of crossbred dairy cattle in smallholder farms in Kenya. *SpringerPlus,* 5, 63.

23. Infonet-Biovison. "Cattle Breeds and Breeding." 2017: http://www.infonet-biovision.org/AnimalHealth/cattle-breeds-and-breeding (accessed November 9 2017)

24. Heuzé V., Tran G., Hassoun P., Brossard L., Bastianelli D., Lebas F., 2015. *Cotton seeds*. Feedipedia, a programme by INRA, CIRAD, AFZ and FAO. https://www.feedipedia.org/node/742 *Last updated on May 12, 2015, 14:25*

25. Alemu, W., Melaku, S. & Tolera, A. (2010). Supplementation of cottonseed, linseed, and noug seed cakes on feed intake, digestibility, body weight, and carcass parameters of Sidama goats. *Trop. Anim. Health Prod.*, 42, 623–631.

26. Tedeschi, L. O. (2006). Assessment of the adequacy of mathematical models. *Agric. Syst.*, 89, 225-247.

**Appendix S2: Rangeland Production Model Description**

## Summary

The purpose of the beta Rangeland Production model is to quantify the potential of a given site to support ruminant livestock and to support wildlife populations, given local environmental and management conditions.

## The Model

### How it Works

The model consists of two dynamic and interacting submodels: a pasture production submodel and a ruminant diet and physiology submodel. The pasture production submodel is the Century model (version 4.6, [1]); this model uses climate and soils data to predict grass growth. The herbivore submodel simulates diet selection from among the available grass types and estimates whether the selected diet meets or exceeds maintenance energy and protein needs, including pregnancy and lactation energy requirements for females. The herbivore diet and physiology model is adapted from GRAZPLAN, which was developed for ruminant livestock [2]. While the Century 4.6 executable is called as-is from the rangeland production model, only selected aspects of the GRAZPLAN herbivore physiology model were adapted. CENTURY is fully documented elsewhere ([1], [3], and citing articles), but full equations for the animal submodel adapted from GRAZPLAN appear in the relevant submodel sections below.

The following model description follows the Overview, Design Concepts and Details protocol of [4], [5].

### Entities, state variables and scales

Model inputs are listed in Table 1. The model is not an agent-based model but instead consists of interacting populations that are modeled as entities. A livestock herd is composed of one or multiple age/sex classes, and each age/sex class is a model entity characterized by its breed, sex, average age (days), average weight (kg), weight at birth (kg), and standard reference weight (kg). The standard reference weight (cf. [2]) is the weight of a mature female in median condition and varies by breed (Table 2). Additional inputs for male animals include castrate

status (castrate or entire), and additional inputs for breeding females include the average month of conception, average duration of lactation, and average calving interval, in months (i.e., months between successive births for the average breeding cow).

Table 1. Required data inputs for the beta Rangeland Production model.

| **Category** | **Dataset** | **Units or allowable values** |
|---|---|---|
| Soil | Percent sand, silt, clay | percent |
| | Bulk density | g per $cm^3$ |
| | pH | pH scale |
| Climate | Monthly average minimum daily temperature | degrees C |
| | Monthly average maximum daily temperature | degrees C |
| | Monthly precipitation | cm |
| Site | Site steepness | ordinal value between 1 and 2 |
| | Latitude | degrees |
| | Biome Century parameter set | E.g., arid shrubland, C3 grassland, C4 grassland |
| | Site management history | May be calculated via back-calculation if empirical biomass is known |
| | Site area | ha |
| Forage | Forage Century parameter set | e.g., C3 grass, C4 grass, temperate grass and clover |
| | Digestibility | optional |
| | Crude protein content | optional |
| | N multiplier | optional |
| Livestock for each age/sex class | General breed | *B. indicus, B. taurus* |
| | Weight at birth | kg |
| | Stocking density | animals per ha |
| | Sex | Entire male, castrate male, breeding female, non-breeding female |
| | Age | days |
| | Conception month (for breeding females) | Month relative to model |

| | | |
|---|---|---|
| | | starting month (may be negative; must be smaller than calving interval) |
| | Calving interval (for breeding females) | months |
| | Lactation duration (for breeding females) | months |

Table 2. Standard reference weights (SRW) for typical cattle breeds. The standard reference weight gives the weight in kg of a mature female in median condition and must be supplied by the user. The values here were adapted from Table 1.12 in [6] and are provided as guidance for users.

| **Breed** | **SRW (kg)** | **General breed** |
|---|---|---|
| Chianina | 700 | *Bos taurus* |
| Charolais, Maine Anjou, Simmental | 650 | *Bos taurus* |
| Angus, Blond d'Aquitane, Brahman, Brahman x Hereford, Hereford, Murray Grey, Limousin, Lincoln Red, Friesian, South Devon | 550 | *Bos indicus* |
| Shorthorn, Red Devon, Galloway, Red Poll | 500 | *Bos taurus* |
| Ayrshire, Guernsey, AMZ, Sahiwal | 450 | *Bos taurus* |
| Jersey | 400 | *Bos taurus* |

The intended use of the model is to assess regional scale productivity of natural ecosystems, and the impact of animal grazing on those ecosystems. The model is not intended to be used to assess detailed management scenarios. Therefore the modeled livestock herd is characterized in a general way and is static throughout a model run. At each model step, the animal physiology submodel calculates the maintenance energy and protein requirements of each livestock class; these reflect the animal's age, weight, and general characteristics such as breed and normal size. Because the energy requirements of breeding females fluctuate greatly during the reproductive cycle [7], the model tracks reproductive status and additional energy requirements due to pregnancy or lactation for these females. The reproductive cycle itself is specified by the user from input describing the average calving interval (i.e., the total length of

the reproductive cycle in months) and the average conception month (i.e., month relative to the modeled time period that conception occurs). The model calculates total metabolizable energy intake from the diet according to its biomass and digestibility, and diet sufficiency is recorded via comparison of metabolizable energy intake versus energy requirements.

When breeding females are included in the modeled herd, cows undergo cycles of conception, pregnancy, and lactation because these reproductive stages have strong influences on energy demands of the herd [7]. The model does not estimate allocation of the diet to growth above maintenance requirements, but instead simply records whether maintenance needs were met or exceeded. Therefore most animal state variables, including age and weight, are static; only reproductive status of breeding females and its impact on energy and protein requirements is updated.

The model accounts for fluctuations in forage demand due to cycles of conception, pregnancy, and lactation by calculating energetic requirements of pregnancy and lactation for breeding females. From inputs supplied by the user that specify conception month, lactation duration, and calving interval, the model calculates for each time step whether the breeding female class is currently pregnant, lactating, or open (i.e. not pregnant and not lactating). The model does not simulate herd dynamics. Births and deaths are not tracked, and suckling calves are assumed to receive nutrition only from milk.

Grass types, representing forage resources, are also model entities; the model may include as many different grass types as are supplied by the user. Each grass type is characterized by a set of Century parameters describing its growth pattern, response to stress, etc. Century reports aboveground live and standing dead vegetation at each time step. Because these are expected to be of greatly different nutritional value for herbivores [8], live and dead portions of each grass type are characterized by different quality parameters. Each forage class is characterized by its current biomass (kg/ha), dry matter digestibility (DMD; %), crude protein content (%), and initial relative biomass (%). The biomass of each forage class fluctuates at each model step as the grass grows and is consumed by herbivores.

Digestibility is a crucial quantity impacting both intake and digestion of forage by livestock [8]. The digestibility of live and dead matter for each forage class may be supplied by the user, if known, or optionally may be calculated at each time step by the model from crude

protein concentration. This calculation follows the regression equations published by [9] for perennial African grasses.

The model is "point-based" with all units being per hectare, so it can be interpreted to represent the dynamics of one ha of pasture. The model operates on a monthly time step.

## Process overview and scheduling

The model is initialized with user input specifying the number of grass types existing in the pasture and their relative initial biomass (Fig. 1). Because Century can simulate only one grass type at a time, a parallel Century simulation is run for each grass type. These simulations are initialized with a "spin-up" period including a hypothetical management scheme typical for the region (see "estimated management history" below). Following the spin-up, the user-supplied initial relative biomass is used to calculate relative abundance of all grass types. Total site biomass is calculated as the weighted average of the biomass reported by Century of each grass type, where each type is weighted by its user-supplied initial % biomass. The biomass of live and standing dead vegetation is calculated in proportion to their relative biomass reported by Century.

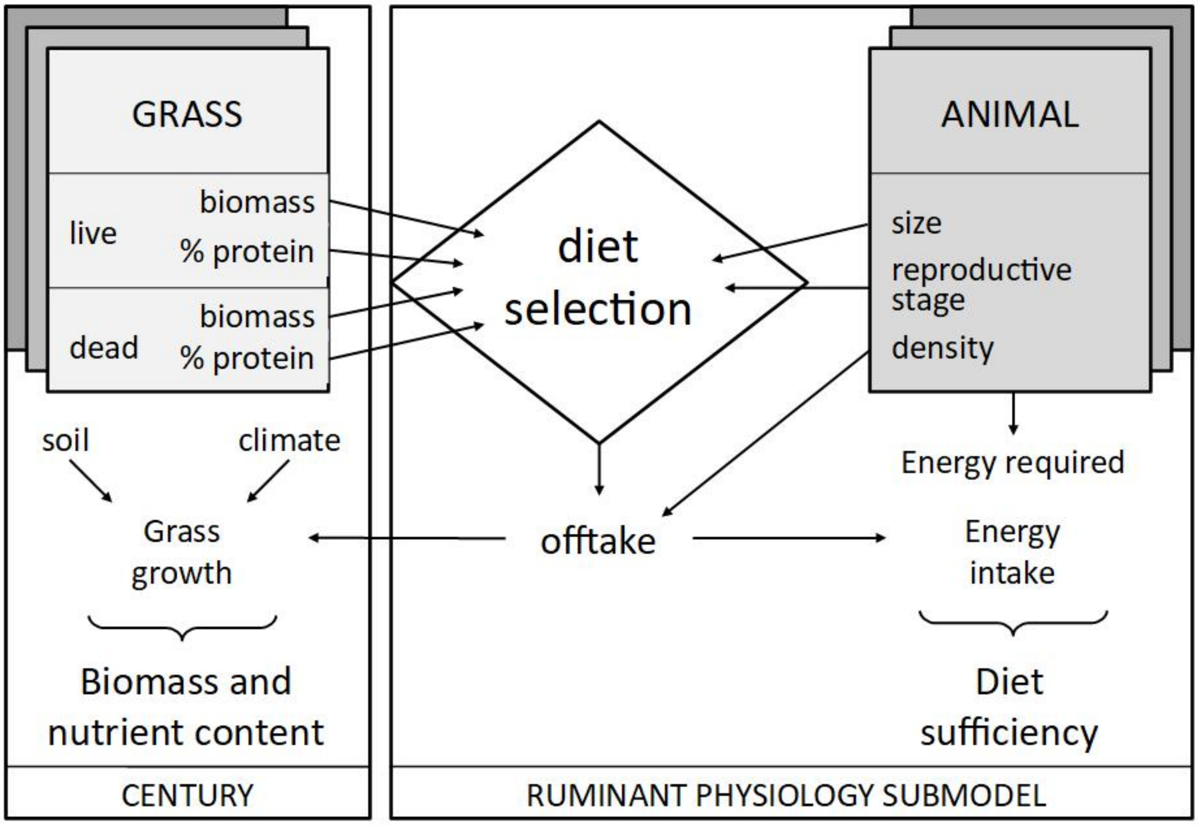


Figure 1. Model overview, showing coupled integration of the Century model (left), which predicts grass growth and nutrient cycling, and the ruminant physiology model adapted from GRAZPLAN (right), which predicts diet selection and diet sufficiency.

The user is required to specify a management threshold, representing the minimum required biomass to be left standing after livestock diet selection. The management threshold does not describe carrying capacity *per se*, but instead is a modeling artifact that is required for boundary cases where simulated densities are high relative to the productivity of the site. In a case when livestock demand for forage is greater than the management threshold would allow, intake of forage is restricted to leave residual biomass equal to the management threshold. It is expected that in this case, the restricted diet would be insufficient to meet maintenance requirements.

The diet selection submodel simulates selective feeding by each herbivore class among forage classes (i.e., aboveground live and standing dead portions of each grass type). For details,

see “Diet selection”, in “Submodels”, below. When there are multiple herbivore types present in the simulation, the diet selected by each herbivore class is calculated separately from the available forage. If the sum of forage biomass selected by herbivores exceeds available forage according to the management threshold, the intake of each herbivore class is reduced proportional to its demand so that forage consumed is equal to available forage (following [10]). This rule is applied separately to each grass type, meaning that if demand for one grass type exceeds availability, intake of that grass type may be decreased while intake of other grass types remains constant. In this situation, the relative proportions of different grass types would differ from what was selected according to the diet selection submodel.

After the calculation of diet selected by each herbivore class and potential restriction of the diet according to the management threshold, energy and protein contents of the diet are compared to maintenance energy and protein requirements for maintenance, including pregnancy and lactation for breeding females (see “Maintenance requirements”, in “Submodels”, below).

Century includes pre-parameterized grazing events that impact ecosystem function through removal of live and dead biomass, return of nutrients to the soil via feces and urine, alteration of the root:shoot ratio, and altered N content of live shoots and roots [11]. After completion of diet selection, offtake of each grass type is formatted as a grazing event in Century using the removal of biomass calculated by diet selection so that impacts of grazing are reflected in grass growth in the next model step. A “template” grazing event parameterization must be supplied by the user to specify other impacts of grazing beyond biomass removal. Several template levels are pre-supplied in Century sample inputs and differ principally in terms of their impact on grass growth: for example, in the “GL” template level, grass growth rate is unrelated to grazing intensity, while in the “GH” template level, grazing intensity affects grass growth in a humped-shape relationship (the “grazing-tolerant” system described by [11]).

The percent of biomass removed from each forage class is calculated and supplied as input to Century for the active month. This input is applied by Century in calculation of growth for the next month. In the next step of the model, percent growth of each forage class is calculated from Century outputs and applied to calculate forage available for diet selection in the next month.

## Initialization

The initialization of most model quantities is controlled by user input. Soil and aboveground nutrient pools in Century are established through a 3000-year long "spin-up" period. During this period a hypothetical historical management regime is applied (see Back-calculation of management, in "Submodels", below).

Several parameters in the livestock physiology submodel vary by breed, reflecting differences between temperate and tropical breeds in their tolerance of heat, ability to metabolize low quality forages, etc [2]. These parameters apply to the broad categories of B. taurus (temperate breeds), B. indicus (tropical breeds), and indicus X taurus breeds only. The user must specify whether livestock belong to B. taurus, B. indicus, or indicus X taurus cross.

The livestock birth weight is used to predict many quantities in the animal physiology submodel [2]. Although there is considerable variation between livestock breeds in this quantity, this variation is complex and is related to sire vs dam breed [12]. If the user does not supply a birth weight, it is assumed that such information is not known and birth weight is equal to the average of all values given in ([12] Table 1), or 34.7 kg.

## Input

The necessary user input is described below under Data Needs.

## Submodels

While full model equations for routines of the livestock diet selection and physiology submodel are included below, we have not reproduced full documentation for the Century model because it can be found elsewhere (cf. [3] and citing articles). Fixed parameters for the livestock submodel refer to animal type and are prefixed by "C", following [2]; see Table 3 for all fixed parameter values.

### Plant growth and nutrient cycling

These submodels are a part of the Century model. See [3] for details.

### Removal of plant material by herbivores

These submodels are a part of the Century model. See [3] for details. Nutrient deposition by herbivores is included in Century's representation of grazing. The percent of

biomass density removed by herbivores is calculated directly from the diet selection submodel and supplied to Century as “flgrem” and “fdgrem” grazing parameters, denoting percent removal of standing live and dead vegetation, respectively.

**Diet selection**

The diet selection submodel was adopted from the GRAZPLAN model [2] and describes the diet selected by a ruminant animal from available forage. The diet is selected on the basis of relative ingestibility and relative availability of each forage class, until the maximum intake for that herbivore class is reached. Maximum intake ($I_{max}$, kg dry matter eaten per day; equation 1) is calculated primarily from the animal’s standard reference weight (*SRW*) and current size relative to *SRW*, *Z* (equation 2). Relative size (*Z*) is calculated from the normal weight (*N*), which is defined by the animal’s current weight (*W*) and age (*A;* equations 3, 4). The maximum intake is also impacted by a correction factor for animals in high relative condition (*CF*; equation 5), and by a lactation factor (*LF*) for lactating females. The correction factor is calculated from body condition (*BC*; equation 6), the ratio of the animal’s current weight to its normal weight.

The lactation factor (equations 7-11) describes the increased maximum intake of a lactating female and is based on a lactation curve driven by the age of the calf ($A_{young}$). The factor *LA* is related to the cow body condition (equation 9); in the original GRAZPLAN formulation, this was related to body condition at parturition (cf. equation 10 in [2]), but we have simplified this factor to depend on a static body condition score. Similarly, the factor *LB* (equation 10) is based on the lactating animal’s body condition relative to the age of the calf. It is assumed that all lactating females are suckling one calf.

$$I_{max} = CI_1 \times SRW \times Z \times \left(CI_2 - Z\right) \times CF \times LF \quad (1)$$

where

$$Z = \frac{N}{SRW} \quad (2)$$

$$N = CN_3 \times N_{max} + \left(1 - CN_3\right) \times W \quad (3)$$

$$N_{max} = SRW - \left(SRW - W_{birth}\right) \times \exp\left(\frac{-CN_1 \times A}{SRW^{CN_2}}\right) \quad (4)$$

$$CF = BC \times \frac{CI_{20} - BC}{CI_{20} - 1} \quad (5)$$

$$BC = \frac{W}{N} \quad (6)$$

$$LF = 1 + CI_{19} \times M_i^{CI_9} \times \exp \quad (7)$$

where

$$M_i = \frac{A_{young}}{CI_i} \quad (8)$$

$$LA = 1 - CI_{15} + CI_{15} \times BC \quad (9)$$

Following the calculation of maximum potential intake, the diet selection routine predicts what proportion of the potential intake is selected from each class of forage available. In a model run where one grass type is simulated, two forage classes will always be available: the live fraction, and the standing dead fraction of that grass type. When two or more grass types are simulated, the number of available forage classes is equal to two times the number of grass types simulated. In the equations below, the subscript "d" indicates one forage class.

Unlike the GRAZPLAN model, which divides available forage into 6 fixed-digestibility classes (cf. [2], p. 6), the Rangeland model calculates availability and digestibility directly from live and dead fractions of available forage as reported by Century. Each forage class is characterized by its biomass (*B;* kg/ha), crude protein content (*P;* g * $g^{-1}$), digestibility (*DMD;* 0 - 1), species factor (*SF* = 0 for C3 grasses, *SF* = 0.16 for C4 grasses), and the proportion of available biomass that is represented by this forage type ($\phi$, 0 - 1). The proportion of total forage represented by legumes, by weight ($\phi_{legume}$, 0 - 1), influences intake of all forage classes.

Prior to beginning diet selection, the forage classes are sorted according to their digestibility; the calculation of the proportion of potential intake that is selected from each class (equations 10-17) is applied first to the most digestible class, and then to the next most digestible, etc. At each application of equations 10-17 to a forage class, the unsatisfied capacity ($UC_d$) tracks the proportion of potential intake that is not yet satisfied by selection of previous,

more digestible, forage classes. This simulates the preferential selection of high-digestibility forage classes before classes of lower digestibility.

For each forage class, the proportion of potential intake selected from this class ($R_d$, equation 10) is a product of its "relative availability" and its "relative ingestibility". Relative availability ($F_d$, equation 11) is a product of the unsatisfied capacity according to selection of more digestible forage classes, the predicted rate of eating this forage class ($RR_d$, equation 13) and the relative time spent eating this forage class ($RT_d$, equation 17). $RR_d$ and $RT_d$ are impacted by the relative height of the forage class, $HR_d$ (equation 15) which is calculated with the assumption that complete cover of one forage class corresponds to a forage height of 3 cm. The rate of eating and time spent eating are also impacted by the size of the animal, reflected in the size factor, $ZF_d$ (equation 16; see equation 2 for the calculation of the animal's current relative size, $Z$).

$$R_d = F_d \times RQ_d \times \left(1 + CR_2 \times \left(\sum_{d=1}^{N} F_d\right) \times \phi_{legume}\right) \quad (10)$$

where

$$F_d = UC_d \times RR_d \times RT_d \quad (11)$$

$$UC_d = max\left(0, 1 - \sum_{d=1}^{d-1} F_d\right) \quad (12)$$

$$RR_d = 1 - \exp\left(-\left(1 + CR_{13} \times \phi_d\right) \times CR_4 \times HF_d \times ZF \times B_d\right) \quad (13)$$

$$HF_d = 1 - CR_{12} + CR_{12} \times HR_d \quad (14)$$

$$HR_d = 0.003 \times \quad (15)$$

$ZF = 1 + \left(CR_7 - Z\right)$ if $Z < CR_7$, else

$$ZF = 1 \quad (16)$$

$$RT_d = 1 + CR_5 \times \exp \quad (17)$$

The "relative ingestibility" of a forage class ($RQ_d$) is calculated primarily from its digestibility (equation 18). The species factor ($SF_d$) reflects higher predicted intake of C4 grasses than C3 grasses of similar digestibility (2).

$$RQ_d = 1 - CR_3 \times \left(CR_1 - \left(1 - \phi_{legume}\right) \times SF_d - DMD_d\right) \quad (18)$$

The actual intake of each forage class, $I_d$ (kg per ha per day), is calculated from the proportion of maximum intake selected from each class (equation 19), and the total intake of forage is calculated as the sum across classes. The average digestibility of the diet, $DMD_f$, is calculated from the digestibility of each forage class and its proportion of the diet (equation 21); similarly, the total crude protein intake of the diet, $CPI_f$, is calculated from crude protein content of each forage class (equation 22).

$$I_d = I_{max} \times R_d \tag{19}$$

$$I_f = \sum_{d=1}^{N} I_d \tag{20}$$

$$DMD_f = \frac{\sum_{d=1}^{N} \left( I_d \times DMD_d \right)}{I_f} \tag{21}$$

$$CPI_f = \sum_{d=1}^{N} \left( I_d \times CP_d \right) \tag{22}$$

**Supplemental feed**

The Rangeland model is meant to be applied to extensive grazing applications where the majority of animals' diet is comprised of forage. The GRAZPLAN model includes the ability to simulate supplemental feeding, and for the purposes of testing we have included some of this functionality. If supplemental feed, such as concentrates, is available, its intake is predicted according to the relative digestibility of the supplement relative to the available forage classes. Therefore the model assumes that an animal will select supplement before selecting forage of equal or lower digestibility. The predicted proportion of maximum intake selected from supplement is calculated similarly to the predicted intake of each forage class, according to the supplement's ingestibility and energy content (equations 23 - 25). The ingestibility of the supplement ($RQ_s$) is calculated from its digestibility, while its energy content (the ratio of metabolizable energy to dry matter, $M/D_s$) must be supplied by the user. The total intake of supplement, $I_s$, is calculated similarly to the total intake of forage (equation 26). After selection of supplement, before diet selection proceeds on forage classes of equal or lesser digestibility, unsatisfied capacity (*UC*) is decreased by $F_s$.

$$R_s = F_s \times RQ_s \tag{23}$$

where

$$F_s = min\left(\frac{\frac{DMO_s}{I_{max}}}{RQ_s}, UC, \frac{CR_{11}}{M/D_s}\right) \tag{24}$$

$$RQ_s = 1 - min\left(CR_{14}, \left(CR_3 \times \left(CR_1 - DMD_s\right)\right)\right) \tag{25}$$

$$I_s = I_{max} \times R_s \tag{26}$$

Once diet selection completes, if protein content of the diet is low, maximum intake is reduced and diet selection is recalculated once according to the adjusted maximum intake. The reduction factor for maximum intake is calculated from the total intake of rumen degradable protein, *RDPI*, and the requirement for rumen degradable protein, *RDPR* (equation 27). For cattle of *B. indicus* breeds, this reduction factor is multiplied by 0.5; for cattle of *B. indicus* cross breeds, the reduction factor is multiplied by 0.75.

Rumen degradability of crude protein of supplement, $dg_s$, which is used to calculate intake of rumen degradable protein from supplement, is required user input. Relative feeding level, *L*, describes the ratio of maintenance energy requirements that are supplied by the diet selected and is described in equation 40 in the description of Maintenance protein and energy requirements, below.

$$I_{max,reduced} = I_{max} \times \frac{RDPI}{RDPR} \tag{27}$$

where

$$RDPI = RDPI_f \times \left(1 - CRD_1 - CRD_2 \times DMD_f\right) \times L + RDPI_s \times \left(1 - CRD_3 \times L\right) \tag{28}$$

$$RDPI_f = CPI_f \times min\left(0.84 \times DMD_f + 0.33, 1\right) \tag{29}$$

$$RDPI_s = CP_s \times dg_s \times I_s \tag{30}$$

**Energy and protein content of the diet**

After completion of a maximum of two iterations of diet selection, the protein and energy content of the diet are calculated. The total metabolizable energy content of the diet, $MEI_{total}$, is the sum of metabolizable energy from forage in the diet and from supplement, if supplement was

included (equations 31-33). Calculation of metabolizable energy from supplement, $MEI_s$, requires input from the user describing ether extract of the supplement ($EE_s$, g per g).

$$MEI_{total} = MEI_f + MEI_s \quad (31)$$

where

$$MEI_f = (17 \times DMD_f - 2) \times I_f \quad (32)$$

$$MEI_s = (13.3 \times DMD_s + 23.4 \times EE_s + 1.32) \times I_s \quad (33)$$

Protein content of the diet is defined as protein available for maintenance and growth, which is summarized in GRAZPLAN [2] as digestible protein leaving the stomach (*DPLS*). This is calculated from digestible undegraded protein and digestible microbial crude protein (equation 34).

$$DPLS = D_{udp} \times UDPI + DPLS_{mcp} \quad (34)$$

where

$$D_{udp} = max\left(CA_1, min\left(CA_3 \times CPI_f - CA_4, CA_2\right)\right) \quad (35)$$

$$UDPI = (I_s \times CP_s - RDPI_s) + (CPI_f - RDPI_f) \quad (36)$$

$$DPLS_{mcp} = CA_6 \times CA_7 \times RDPR \quad (37)$$

**Maintenance energy and protein requirements**

At each modeled timestep, total maintenance metabolizable energy (*ME*) requirements are calculated as the sum of ME requirements for maintenance, and for breeding females, maintenance requirements for pregnancy ($ME_c$) or lactation ($ME_l$; equation 38). The requirement of energy for maintenance is the sum of basal metabolic energy requirements and the energy requirements of grazing (equation 39). The energy requirements of grazing include an estimation of the distance walked per day (*D*, equation 44), which is calculated from the biomass of forage, the total stocking density of animals across age and sex classes (*sd*), and a site steepness score (*S*) ranging from 1-2. The efficiency of energy use for maintenance, $k_m$, is calculated from the energy content of forage in the diet ($M/D_f$, equations 45-46). For male animals, *MEm* is multiplied by 1.15.

$$ME_{total} = ME_m + ME_c + ME_l \quad (38)$$

$$M\,E_m = \frac{E_{metab} + E_{graze}}{k_m} + C\,M_1 \times ME\,I_{total} \quad (39)$$

$$L = \frac{ME\,I_{total}}{M\,E_m} - 1 \quad (40)$$

where

$$E_{metab} = C\,M_2 \times W^{0.75} \times max\left(\exp\left(C\,M_3 \times A\right), C\,M_4\right) \quad (41)$$

$$E_{graze} = C\,M_6 \times W \times I_f \times \left(C\,M_7 - DM\,D_f\right) + E_{move} \quad (42)$$

$$E_{move} = C\,M_{16} \times D \times W \quad (43)$$

$$D = \frac{S \times min\left(1, C\,M_{17}/sd\right)}{C\,M_8 \times B_{\text{green}} + CM_9} \quad \text{if } B_{green} > 100 \text{ kg ha}^{-1}\text{, else}$$

$$D = \frac{S \times min\left(1, C\,M_{17}/sd\right)}{C\,M_8 \times B_{dead} + C\,M_9} \quad \text{if } B_{green} < 100 \text{ kg ha}^{-1} \text{ and } B_{dead} > 100 \text{ kg ha}^{-1}\text{, else}$$

$$D = 0 \quad (44)$$

$$k_m = C\,K_1 + C\,K_2 \times M/D_f \quad (45)$$

$$M/D_f = \frac{ME\,I_f}{I_f} \quad (46)$$

Energy requirements of pregnancy are derived from the body weight of the cow for pregnancy purposes, *BW*, and the relative age of the fetus, *RA* (equation 47). The energy requirements of pregnancy are simplified from the implementation of [2] in two ways: first, the body condition of the fetus ($BC_{foet}$) is assumed to be 1; that is, the fetus is assumed to be in median condition. Second, we also assume that each pregnant cow is pregnant with one fetus.

$$ME_c = \frac{\left(C\,P_8 \times C\,P_5 \times BW \times \frac{C\,P_9 \times C\,P_{10}}{C\,P_1} \times \exp\left(C\,P_{10} \times \left(1 - RA\right) + C\,P_9 \times 1 - \exp\left(C\,P_{10} \times \left(1 - RA\right)\right)\right)\right)}{C\,K_8} \quad (47)$$

where

$$BW = \left(1 - C\,P_4 + C\,P_4 \times Z\right) \times C\,P_{15} \times SRW \quad (48)$$

$$RA=\frac{A_{foet}}{CP_1} \tag{49}$$

Energy requirements of lactation (equation 50) are related to the body condition of the cow and the age of the calf; they have also been simplified from [2]. All lactating cows are assumed to be suckling one calf. While maximum milk production is calculated (*$MP_{max}$,* equation 51), the actual production of milk is not limited by insufficient intake of ME or by the suckling young's ability to consume milk.

$$ME_l=\frac{MP_{max}}{CL_5\times k_l} \tag{50}$$

$$MP_{max}=CL_0\times SRW^{0.75}\times Z\times BC\times M_m^{CL_3}\times \exp\left(CL_3\times\left(1-M_m\right)\right) \tag{51}$$

$$M_m=\frac{A_{young}+CL_1}{CL_2} \tag{52}$$

Total maintenance requirements for protein are similarly calculated as the sum of basal protein requirements, and protein requirements of pregnancy or lactation for breeding females (equation 53). Basal protein requirements (equation 54) are derived from the animal's body weight and intake of forage and supplement.

$$P_{total}=P_m+P_c+P_l \tag{53}$$

$$P_m=CM_{12}\times\ln\left(W\right)-CM_{13}+CM_{10}\times\left(I_f+I_s\right)+CM_{14}\times W^{0.75} \tag{54}$$

Protein requirements of pregnancy (equations 55-57) are calculated similarly to energy requirements of pregnancy and are primarily related to the body weight of the cow for pregnancy purposes (*BW*) and the relative age of the fetus (*RA*).

$$P_c=P_{c1}\times P_{c2} \tag{55}$$

$$P_{c1}=CP_{11}\times CP_5\times BW\times\frac{CP_{12}\times CP_{13}}{CP_1} \tag{56}$$

$$P_{c2}=\exp\left(CP_{13}\times\left(1-RA\right)+CP_{12}\times\left(1-\exp\left(CP_{13}\times\left(1-RA\right)\right)\right)\right) \tag{57}$$

Protein requirements of lactation (equation 58) are, like the energy requirements of lactation, calculated from predicted maximum milk production without limiting by cow body condition or calf suckling ability.

$$P_l = CL_{15} \times \frac{MP_{max}}{CL_6} \quad (58)$$

**Back-calculation of management**

The Century model requires a long "spin-up" period to establish soil nutrient pools, and a management schedule must be supplied for this period. The required management schedule consists of scheduled grazing events (months when there were herbivores present) where each grazing event is associated with a grazing intensity level (percent biomass removed by herbivores). This presents both a problem, in that the management history of a site is very rarely known in the detail that must be supplied to the model, and an opportunity. This *back-calculate management* routine adjusts the simulated grazing schedule for a period of time prior to an empirical biomass measurement until the simulated biomass matches the empirical measurement. The calculated schedule can then be compared to any known management history for the site as a check on the model's ability to simulate local biomass dynamics.

Given a single empirical biomass measurement and the date it was taken, the routine modifies scheduled grazing events either by adding or removing grazing events, modifying the intensity of grazing events, or both. The routine runs Century up to the empirical measurement point and if simulated biomass differs from empirical by more than a user-supplied target threshold, grazing events are added or removed to the simulation and Century is run again. If the user specifies that the schedule of grazing alone should be modified, grazing events are added or removed from the schedule one month at a time. Grazing events are added or removed first from the month immediately preceding the empirical measurement date, proceeding backwards in time with each iteration to the maximum number of years prior to the measurement date that may be modified (a user-supplied time limit). If intensity alone should be modified, the routine modifies the grazing parameter definition file by adding or subtracting 10% from the "flgrem" parameter, the amount of live biomass removed. The amount of standing dead biomass removed (the "fdgrem" parameter) is calculated as 10% of flgrem [11].

If the user specifies that both the schedule and intensity should be modified, the routine first modifies the schedule until no more opportunities exist to do so, and then modifies intensity. For example, if simulated biomass is higher than empirical biomass, the routine adds grazing events prior to the measurement date. If grazing events are added to every month within the maximum amount of time allowable to modify but simulated biomass is still higher than empirical, the routine then begins to increase grazing intensity. Intensity is then modified at each iteration until the target threshold is met or the maximum allowable iterations are completed.

Required user inputs for this submodel include an empirical biomass measurement and the date (year and month) of its measurement; whether this measurement relates to total standing biomass, or live biomass only; an existing schedule file; how many years prior to the empirical measurement date to potentially manipulate; maximum iterations to undertake; a threshold tolerance (the routine completes if this difference between simulated and empirical biomass is under this tolerance); and whether to vary the schedule of grazing events, the intensity of grazing events, or both.

The schedule file supplied as initial input must include a schedule block containing the empirical measurement date and the maximum number of years prior to empirical measurement that may be manipulated, in a non-repeating sequence. If this condition is not met, the routine stops with an error. Note that when grazing intensity is modified, all grazing events in the schedule are affected, not just those immediately prior to the empirical measurement date.

## Model outputs

The model is run for a fixed amount of time, which is given by the user as input. The model produces a time series as output which contains, for each time step, the biomass of each grass type, the offtake selected by each animal type, and for each animal type, total energy and protein required and total energy and protein intake from the diet. From these quantities, the user may calculate the ratio of energy intake to maintenance energy requirements in that timestep, indicating whether the diet was sufficient to maintain the animal’s weight and reproductive status.

## Limitations and Simplifications

The model is designed to be applicable in multiple climatic zones and regions of the world; this generality implies necessary simplifications that distinguish the model from other existing models that are much more complex and site-specific (e.g., SPUR [13], GrassGro [14], EcoMod [15]).

Each forage type is modeled with an independent Century simulation; therefore interactions such as competition and facilitation between plant types are not included in this model. However, plant types can exhibit indirect facilitation through herbivore diet selection, such that preference for one plant type will free the other from consumption.

Our dependence on pre-parameterized plant types already developed for the Century model means that we cannot model the effects of improved forage types that may be associated with greatly improved feed conversion efficiency over natural grass types [16]. Though the option exists for the user to override model-generated values for crude protein and digestibility, it is currently unknown what other parameter changes would be necessary to better reflect the growth and senescence of improved grass strains.

We assume that the livestock herd consists of adult animals only and do not model population dynamics such as births or deaths. While energy demand of pregnancy and lactation are accounted, the number of animals in each sex and age class does not change. We assume that all cattle are of beef cattle type, thus ignoring slightly modified parameter values provided in [2] that apply to lactating cows of dairy type. During pregnancy, we do not track the weight of the fetus explicitly, but assume that it is in median condition for its stage of development.

The model represents a single area or location and does not include movement of animals between pastures; it also does not count importation of supplemental feed above what the simulated forage growth provides. The model is therefore limited to extensive grazing systems and short time scales where these restrictions are acceptable.

Table 3. Fixed parameter values for the livestock physiology submodel, adapted from [2]. For those parameters whose value differs between cattle of breed *B. indicus* v *B. taurus,* the values are given in column A (for *B. indicus*), B (for *B. taurus*) or C (for *indicus* x *taurus* crosses).

| Parameter | Description | Units | Value | Value A | Value B | Value C |
|---|---|---|---|---|---|---|
| $CN_1$ | Growth rate constant | kg $^{0.27}$ $d^{-1}$ | 0.0115 | | | |
| $CN_2$ | Allometric scalar for growth rate | | 0.27 | | | |
| $CN_3$ | Weighting factor for slow growth | | 0.4 | | | |
| $CI_1$ | Relative size | kg $kg^{-1}$ | 0.025 | | | |
| $CI_2$ | Rumen development | $d^{-1}$ | 1.7 | | | |
| $CI_8$ | Lactation: peak intake time | $d^{-1}$ | 62 | | | |
| $CI_9$ | Intake curvature | | 1.7 | | | |
| $CI_{10}$ | dairy cow factors | $kg^{-1}$ | 0.6 | | | |
| $CI_{11}$ | body condition loss | | 0.05 | | | |
| $CI_{19}$ | peak intake level: 1 young | | 0.416 | | | |
| $CI_{20}$ | Effect of body condition | | 1.5 | | | |
| $CR_1$ | Digestibility: peak | 0-1 | 0.8 | | | |
| $CR_2$ | legume effect | | 0.17 | | | |
| $CR_3$ | slope | | 1.7 | | | |
| $CR_4$ | Availability: grazing rate | $kg^{-1}$ | 0.00078 | | | |
| $CR_5$ | grazing time | | 0.6 | | | |
| $CR_6$ | grazing time | $kg^{-1}$ | 0.00074 | | | |
| $CR_7$ | Relative size on time and rate | | 0.5 | | | |
| $CR_{11}$ | Substitution: supplement M/D | MJ $kg^{-1}$ | 10.5 | | | |
| $CR_{12}$ | Effect of pasture height | | 0.8 | | | |

| | | | | | | |
|---|---|---|---|---|---|---|
| $CR_{13}$ | Effect of proportion in class | | 0.35 | | | |
| $CR_{14}$ | Upper limit on RQ for supplements | | 1 | | | |
| $CK_1$ | $k_m$: M/D in solid diet | | 0.5 | | | |
| $CK_2$ | $k_m$: M/D in solid diet | kg $MJ^{-1}$ | 0.02 | | | |
| $CK_5$ | | | 0.4 | | | |
| $CK_6$ | $k_l$: M/D in solid diet | | 0.02 | | | |
| $CK_8$ | $k_c$ | 0-1 | 0.133 | | | |
| $CK_{13}$ | herbage, zero legume, mid-winter | kg $M^{J-1}$ | 0.035 | | | |
| $CL_0$ | Peak yield scalar if suckling | MJ $kg^{-3/4}$ | 0.375 | | | |
| $CL_1$ | Lactation curve: offset | d | 4 | | | |
| $CL_2$ | peak time | d | 30 | | | |
| $CL_3$ | shape, with young | | 0.6 | | | |
| $CL_5$ | Milk: metabolizability | 0-1 | 0.94 | | | |
| $CL_6$ | energy content | MJ $kg^{-1}$ | 3.1 | | | |
| $CL_{15}$ | Protein content of milk | kg $kg^{-1}$ | 0.032 | | | |
| $CM_1$ | $Me_m$: liveweight gain | | 0.09 | | | |
| $CM_2$ | Basal metabolism: weight scalar | MJ $kg^{-3/4}$ | | 0.31 | 0.36 | 0.335 |
| $CM_3$ | effect of age | d-1 | 8E-05 | | | |
| $CM_4$ | effect of age | | 0.84 | | | |
| $CM_6$ | $E_{graze}$: chewing cost | MJ $kg^{-1}$ | 0.0025 | | | |

| | | | | | | |
|---|---|---|---|---|---|---|
| $CM_7$ | $E_{graze}$: chewing cost | 0-1 | 0.9 | | | |
| $CM_8$ | walking cost | kg $km^{-1}$ | 5.7E-05 | | | |
| $CM_9$ | walking cost | $km^{-1}$ | 0.16 | | | |
| $CM_{10}$ | EFP from solid diet | kg $kg^{-1}$ | 0.0152 | | | |
| $CM_{12}$ | EUP | | | 1.29 E-02 | 1.61 E-02 | 1.45 E-02 |
| $CM_{13}$ | EUP | kg 0.27 $d^{-1}$ | | 3.38 E-02 | 4.22 E-02 | 3.8 E-02 |
| $CM_{16}$ | Energy cost of horizontal walking | MJ $km^{-1}$ $kg^{-1}$ | 0.0026 | | | |
| $CM_{17}$ | Threshold stocking density | head $ha^{-1}$ | 5 | | | |
| $CRD_1$ | Degradability: feeding level | | 0.3 | | | |
| $CRD_2$ | Degradability: feeding level | | 0.25 | | | |
| $CRD_3$ | Degradability: feeding level | | 0.1 | | | |
| $CRD_4$ | RDPR: feeding level | $kg^{-1}$ | 0.007 | | | |
| $CRD_5$ | RDPR: feeding level | $kg^{-1}$ | 0.005 | | | |
| $CRD_6$ | RDPR: feeding level | | 0.35 | | | |
| $CRD_7$ | time of year (forage) | | 0.1 | | | |
| $CA_1$ | UDP digestibility | | 0.05 | | | |
| $CA_2$ | UDP digestibility | | 0.85 | | | |
| $CA_3$ | UDP digestibility | | 5.5 | | | |
| $CA_4$ | UDP digestibility | | 0.178 | | | |
| $CA_6$ | DPLS in MCP | | 1 | | | |

| | | | | | | |
|---|---|---|---|---|---|---|
| $CA_7$ | DPLS in MCP | | 0.6 | | | |
| $CG_2$ | DPLS: efficiency of use for other purposes from solid diet | 0-1 | 0.7 | | | |
| $CG_{12}$ | PCG: reference value | kg $kg^{-1}$ | | 0.092 | 0.072 | 0.072 |
| $CG_{13}$ | PCG: range with maturity at L=1 and BC=1 | kg $kg^{-1}$ | | 0.12 | 0.14 | 0.14 |
| $CP_1$ | Gestation length | d | 285 | | | |
| $CP_4$ | Effect of relative size on birth weight | | 0.33 | | | |
| $CP_5$ | Final conceptus weight:fetus weight | kg $kg^{-}$ | 1.8 | | | |
| $CP_6$ | Conceptus weight | | 2.42 | | | |
| $CP_7$ | Conceptus weight | | 1.16 | | | |
| $CP_8$ | Final conceptus energy content | MJ $kg^{-1}$ | 4.11 | | | |
| $CP_9$ | Conceptus energy | | 343.5 | | | |
| $CP_{10}$ | Conceptus energy | | 0.0164 | | | |
| $CP_{11}$ | Final conceptus protein content | kg $kg^{-1}$ | 0.134 | | | |
| $CP_{12}$ | Conceptus protein | | 6.22 | | | |
| $CP_{13}$ | Conceptus protein | $d^{-1}$ | 0.747 | | | |
| $CP_{15}$ | Normal birth weight:SRW, 1 young | | 0.07 | | | |

# References


1. Parton, W.J., Stewart, J.W. & Cole, C.V. (1988). Dynamics of C, N, P and S in grassland soils: a model. *Biogeochemistry*, 5, 109–131.
2. Freer, M., Moore, A.D. & Donnelly, J.R. (2012). *The GRAZPLAN animal biology model for sheep and cattle and the GrazFeed decision support tool*. CSIRO Plant Industry, Canberra, ACT Australia.

3. Parton, W.J., Scurlock, J.M.O., Ojima, D.S., Gilmanov, T.G., Scholes, R.J., Schimel, D.S., *et al.* (1993). Observations and modeling of biomass and soil organic matter dynamics for the grassland biome worldwide. *Global Biogeochem. Cycles*, 7, 785–809.
4. Grimm, V., Berger, U., Bastiansen, F., Eliassen, S., Ginot, V., Giske, J., *et al.* (2006). A standard protocol for describing individual-based and agent-based models. *Ecol. Modell.*, 198, 115–126.
5. Grimm, V., Berger, U., DeAngelis, D.L., Polhill, J.G., Giske, J. & Railsback, S.F. (2010). The ODD protocol: a review and first update. *Ecol. Modell.*, 221, 2760–2768.
6. CSIRO (1990). *Feeding Standards for Australian Livestock: Ruminants*. Csiro Publishing.
7. IPCC. (2006). Vol. 4: Agriculture, Forestry and Other Land Use. In: *2006 IPCC Guidelines for National Greenhouse Gas Inventories* (eds. Eggleston, H., Buendia, L., Miwa, K., Ngara, T. & Tanabe, K.). IGES, Japan.
8. Coleman, S.W. & Moore, J.E. (2003). Feed quality and animal performance. *Field Crops Research*, 84, 17–29.
9. Illius, A.W., Derry, J. & Gordon, I.J. (1995). Components, processes and dynamics of semi-arid grazing systems. A review of current knowledge. NRI internal discussion document.
10. Pachzelt, A., Rammig, A., Higgins, S. & Hickler, T. (2013). Coupling a physiological grazer population model with a generalized model for vegetation dynamics. *Ecol. Modell.*, 263, 92–102.
11. Holland, E.A., Parton, W.J., Detling, J.K. & Coppock, D.L. (1992). Physiological responses of plant populations to herbivory and their consequences for ecosystem nutrient flow. *Am. Nat.*, 140, 685–706.
12. Comerford, J.W., Benyshek, L.L., Bertrand, J.K. & Johnson, M.H. (1988). Evaluation of performance characteristics in a diallel among Simmental, Limousin, Polled Hereford and Brahman beef cattle. I. Growth, hip height and pelvic size. *J. Anim. Sci.*, 66, 293–305.
13. Foy, J.K., Teague, W.R. & Hanson, J.D. (1999). Evaluation of the upgraded SPUR model (SPUR2.4). *Ecol. Modell.*, 118, 149–165.
14. Moore, A.D., Donnelly, J.R. & Freer, M. (1997). GRAZPLAN: Decision support systems for Australian grazing enterprises. III. Pasture growth and soil moisture submodels, and the GrassGro DSS. *Agric. Syst.*, 55, 535–582.
15. Johnson, I.R., Chapman, D.F., Snow, V.O., Eckard, R.J., Parsons, A.J., Lambert, M.G., *et al.* (2008). DairyMod and EcoMod: biophysical pasture-simulation models for Australia and New Zealand. *Aust. J. Exp. Agric.*, 48, 621–631.

16. Bosire, C.K., Ogutu, J.O., Said, M.Y., Krol, M.S., Leeuw, J. de & Hoekstra, A.Y. (2015). Trends and spatial variation in water and land footprints of meat and milk production systems in Kenya. *Agric. Ecosyst. Environ.*, 205, 36–47.
